# Dynamics of capillary blood flow responses to acute local changes in oxygen and carbon dioxide concentrations

**DOI:** 10.3389/fphys.2022.1052449

**Published:** 2022-12-06

**Authors:** Gaylene M. Russell McEvoy, Brenda N. Wells, Meghan E. Kiley, Kanika K. Kaur, Graham M. Fraser

**Affiliations:** Division of BioMedical Sciences, Faculty of Medicine, Memorial University of Newfoundland, St. John’s, NL, Canada

**Keywords:** microcirculation, oxygen mediated blood flow regulation, capillary, skeletal muscle, exercise, carbon dioxide

## Abstract

**Objectives:** We aimed to quantify the magnitude and time transients of capillary blood flow responses to acute changes in local oxygen concentration ([O_2_]), and carbon dioxide concentration ([CO_2_]) in skeletal muscle. Additionally, we sought to quantify the combined response to both low [O_2_] and high [CO_2_] to mimic muscle microenvironment changes at the onset of exercise.

**Methods:** 13 Sprague Dawley rats were anaesthetized, mechanically ventilated, and instrumented with indwelling catheters for systemic monitoring. The extensor digitorum longus muscle was blunt dissected, and reflected over a microfluidic gas exchange chamber in the stage of an inverted microscope. Four O_2_ challenges, four CO_2_ challenges, and a combined low O_2_ (7–2%) and high CO_2_ (5–10%) challenges were delivered to the surface with simultaneous visualization of capillary blood flow responses. Recordings were made for each challenge over a 1-min baseline period followed by a 2-min step change. The combined challenge employed a 1-min [O_2_] challenge followed by a 2-min change in [CO_2_]. Mean data for each sequence were fit using least-squared non-linear exponential models to determine the dynamics of each response.

**Results:** 7–2% [O_2_] challenges decreased capillary RBC saturation within 2 s following the step change (46.53 ± 19.56% vs. 48.51 ± 19.02%, *p* < 0.0001, *τ* = 1.44 s), increased RBC velocity within 3 s (228.53 ± 190.39 μm/s vs. 235.74 ± 193.52 μm/s, *p* < 0.0003, *τ* = 35.54 s) with a 52% peak increase by the end of the challenge, hematocrit and supply rate show similar dynamics. 5–10% [CO_2_] challenges increased RBC velocity within 2 s following the step change (273.40 ± 218.06 μm/s vs. 276.75 ± 215.94 μm/s, *p* = 0.007, *τ* = 79.34s), with a 58% peak increase by the end of the challenge, supply rate and hematocrit show similar dynamics. Combined [O_2_] and [CO_2_] challenges resulted in additive responses to all microvascular hemodynamic measures with a 103% peak velocity increase by the end of the collection period. Data for mean responses and exponential fitting parameters are reported for all challenges.

**Conclusion:** Microvascular level changes in muscle [O_2_] and [CO_2_] provoked capillary hemodynamic responses with differing time transients. Simulating exercise via combined [O_2_] and [CO_2_] challenges demonstrated the independent and additive nature of local blood flow responses to these agents.

## Introduction

The microcirculation across all tissues is responsible for delivering nutrients, such as oxygen (O_2_), while simultaneously removing waste products such as carbon dioxide (CO_2_), produced from aerobic metabolism (Duling & Berne, 1970; Segal, 2005). Accordingly, it has been well established that blood flow within the microcirculation increases to match local oxygen demand in response to hypoxia or elevated oxidative metabolism during exercise (Hudlicka, 1985; Segal, 2005; Jackson et al., 2010). Under resting and moderate exercise conditions, energy is produced in skeletal muscle *via* oxidative phosphorylation that requires sufficient O_2_ for production of ATP, while simultaneously producing CO_2_ as a waste product. There is a significant positive correlation between the rate of increase in blood flow and the calculated muscle O_2_ uptake (V̇O_2_) at the onset of exercise (Hughson et al., 1996). Extensive data support the principle that increases in blood flow to working muscle during exercise are directly coupled to oxidative demand for production of ATP through vasomotor responses that promote O_2_ transport and delivery (Nuutinen et al., 1982). The dynamics of V̇O_2_ at the onset of exercise, and the accompanying bulk blood flow response, has been a major area of investigation focused on elucidating the limitations to exercise performance, and the underlying mechanisms that govern regional blood flow responses (Murias et al., 2014). At the microvascular level, local changes in arteriolar tone modulate conductance which serves as the key driver of blood flow responses during exercise. Conventional studies that logically employ exercise in humans, or stimulated contractions in animal models, to dynamically increase aerobic muscle metabolism and provoke regional blood flow responses are inherently confounded by a myriad of simultaneous microvascular vasomotor mechanisms. Indeed, multiple metabolic stimuli are integrated within the microcirculation to determine the magnitude and time course of local blood flow (Walløe & Wesche, 1988; Shoemaker and Hughson, 1999; Boushel et al., 2000; Herspring et al., 2008). In the context of exercise, it is difficult to separate the specific contributions that O_2_ and CO_2_ concentrations have on blood flow supply and demand matching from other vasoactive metabolites that regulate blood flow.

Microvascular blood flow responses at the onset of exercise result from the cumulative stimulus of multiple vasoactive stimuli and mechanical factors. Within the first 1-2 muscular contractions, blood flow increases precede changes in V̇O_2_ as measured at the mouth. This sudden increase in blood flow has been attributed to rapid onset vasodilation (Corondilas et al., 1964; Shoemaker and Hughson, 1999; Mihok et al., 2004; VanTeeffelen, 2006; Jackson et al., 2010). Additionally, this increase in blood flow at the arteriolar level increases the shear stress experienced by these vessels, promoting the release of nitric oxide (NO) from the endothelium which stimulates vasodilation in arterioles and feed arteries (Wray et al., 2011). Experimentally it has been shown that the onset of exercise also reduces availability of superoxide, due to an increase in oxygen consumption, leading to an increase in the concentration of NO in the interstitium, resulting in vasodilation (Golub et al., 2014).

Electrically coupled conducted responses have been implicated across multiple levels of microvessels. Changes in electrical potential in both endothelial and vascular smooth muscle cells (VSMC) have been shown to propagate upstream and ultimately modulate arteriolar tone (Cohen et al., 2000; Segal and Jacobs, 2001). Multiple transmembrane channels have been associated with blood flow responses during exercise. Arteriolar VSMC within skeletal muscle depend on Ca^2+^ influx through voltage-gated Ca^2+^ channels and release from internal stores through inositol 1,4,5-triphosphate receptors to regulate myogenic tone (Jackson & Boerman, 2018). During muscle contractions, K^+^ channels located in capillary endothelium are exposed to an accumulation of K^+^ ions and subsequently transduce a signal resulting in endothelial cell hyperpolarization that is transmitted upstream to stimulate arteriole vasodilation (Jackson, 2017).

Exercise decreases the local availability of O_2_ in working skeletal muscle, this increase in demand for O_2_ stimulates an increase in O_2_ supply for metabolic matching. In exercising muscle, increased aerobic metabolism and a lower capillary SO_2_ leads to ATP release from red blood cells (RBC), which is believed to trigger a conducted response that travels upstream causing vasodilation of arterioles and subsequently increase blood flow to areas of muscle with decreased O_2_ availability, thereby matching the high oxygen demand of active skeletal muscle (Jackson, 1987, 2016; Ellis et al., 2012; Ellsworth et al., 2016). An increase in lactate during exercise decreases pH in the muscle; this increase in lactate occurs in heavy exercise, yet muscle blood flow and oxygen uptake increase linearly with work loads (Andersen & Saltin, 1985; Berg et al., 1997). There have been several suggested O_2_ dependent mechanisms involved for local blood flow regulation in active skeletal muscle; however, mechanisms underlying the response to increased CO_2_ have yet to be elucidated.

Changing O_2_ and CO_2_ concentrations in skeletal muscle during exercise have been shown to elicit local blood flow responses, and several potential mechanisms responsible for these responses have been identified. The release of ATP from RBCs has been shown to elicit an increase in blood flow by dilating upstream arterioles providing support for RBCs as both a sensor and stimulus to regulate oxygen concentration in skeletal muscle. RBCs release ATP when hemoglobin desaturates in response to low oxygen environments such as that found in exercise (Jagger et al., 2001; Ellsworth et al., 2016). Arteriolar smooth muscle cells and endothelial cells have also been demonstrated, in *ex vivo* studies, to play a role in O_2_ mediated blood flow responses, however this has not been similarly observed *in vivo* using intravital microscopy methods (reviewed in Jackson, 2016). Furthermore, during exercise, as aerobic metabolism increases, and the rate of CO_2_ production increases proportionally with O_2_ consumption causing elevated tissue partial pressure of carbon dioxide (PCO_2_). Higher tissue CO_2_ concentration on its own, and in combination with the resulting decrease in tissue pH, has been shown to provoke vasodilation in striated muscle (Duling, 1973; Charter et al., 2018).

Targeted manipulation of [O_2_] and [CO_2_] while simultaneously measuring the resulting capillary blood flow response is possible when coupled with intravital video microscopy and precisely controlled gas conditions. The microcirculation is essential in the transport of O_2_ from the blood delivering it into tissue while simultaneously removing CO_2_. Direct manipulation of local O_2_ and CO_2_ concentrations has been previously achieved by employing microfluidic gas exchange chambers that can maintain a constant gas partial pressure in the tissue while simultaneously measuring capillary blood flow in skeletal muscle (Ghonaim, 2011; Ghonaim, 2013; Sové 2021). The use of a gas exchange chamber allows dynamic manipulation of [O_2_] and [CO_2_] both individually and in combination with one another. Previous work manipulating [O_2_] and [CO_2_] have largely focused on steady state flow conditions and as a result, dynamic characterization of capillary level blood flow in response to acute changes in [O_2_] and [CO_2_] have yet to be thoroughly described. Characterizing the time course of [O_2_] and [CO_2_] mediated microvascular blood flow responses is essential to understanding the broader dynamics of conduit artery flow at the onset of exercise. Furthermore, examining the dynamics of microvascular responses under various conditions can provide mechanistic insights which may be masked by overlapping or redundant mechanisms. In this study, we aim to quantify the magnitude and time transient of capillary blood flow responses to acute changes in local [O_2_] and [CO_2_] in skeletal muscle. Additionally, we sought to quantify the additive response to both [O_2_] and [CO_2_] to mimic muscle microenvironment conditions similar to those found following the onset of moderate exercise.

## Materials and methods

### Animal surgery

13 male Sprague Dawley rats (170 g–211 g) were obtained from Charles River Laboratories and were allowed to acclimatize in animal care for 1 week before use. Rats were fed Teklad 2018 (Envigo, Indianapolis, IND, United States) standard rodent chow and allowed water *ad libitum*. All animal protocols were approved by Memorial University Animal Care Committee.

On the day of testing, animals were anesthetized with a 65 mg/kg intraperitoneal injection of sodium pentobarbital (Euthanyl, Bimeda, Cambridge, ON, Canada). Following induction, depth of anesthesia was assessed with palpebral reflex and toe pinch prior to the start of surgery to verify the animal was sufficiently anesthetized. Once in the surgical plane, a rectal temperature probe was inserted to monitor the body temperature of the animal throughout the experiment. Physiological temperatures were maintained between 36–37°C using a heated mat and/or heat lamp as necessary.

The common carotid artery was cannulated to allow continuous monitoring of blood pressure and heart rate (400a Blood Pressure Analyzer, Micro-Med, Louisville, KY, United States). The right jugular vein was cannulated to provide fluid resuscitation (0.5 ml/kg/hr) and maintenance anaesthetic as required. The animal’s heart rate and blood pressure were monitored continuously for variability as well as regular testing the palpebral reflexes and toe pinch to ensure acceptable depth of anaesthesia. Maintenance doses of sodium pentobarbital (22 mg/kg) were administered intravenously when the animal’s mean arterial pressure exceeded 110 mmHg or if the animal responded to adverse stimuli. Animals were tracheotomized and mechanically ventilated (Inspira ASV, Harvard Apparatus, Holliston, MA, United States) with an initial gas mixture of ∼30% O_2_ and 70% N_2_. Respiratory rates and volumes were automatically determined by the ventilator’s built in software based on the animal’s weight. The right extensor digitorum longus (EDL), a muscle of the lower hind limb, was blunt dissected and isolated as previously described (Tyml and Budreau, 1991; Fraser et al., 2012). The distal tendon was cut, the muscle was lifted and cleared from the remaining tissue without damaging the feed artery and vein. The EDL muscle was reflected over the gas exchange chamber on the stage of an inverted microscope (IX73, Olympus, Tokyo, Japan). The EDL was fixed under slight tension at approximately *in situ* length, covered with a polyvinylidene chloride film, bathed in warm saline, and gently compressed with a glass coverslip and microscope slide to isolate the EDL from room air, and to aid in establishing a uniform optical interface that is orthogonal to the incident light path. The animal was allowed to acclimatize on the stage for 30 min following positioning. Following the acclimatization, and with the animal’s body temperature between 36–37°C, an arterial blood sample was collected to measure blood gases (VetScan iSTAT, Abbott Point of Care Inc. Princeton, NJ, United States). Arterial PCO_2_ and PO_2_ were maintained within normal physiological range by adjusting ventilation rate and volume as needed prior to data collection.

The microscopy imaging setup was composed of an Olympus IX73 microscope (Olympus, Tokyo, Japan) fitted for transillumination with a 300 W Xenon light source (Lambda LS-30, Sutter Instruments, Novato, CA, United States). A parfocal beam splitter (Optosplit II Bypass, Cairn Research Ltd. Faversham, United Kingdom) directed light through 420 nm (isosbestic wavelength) and 438 nm (oxygen-sensitive) bandpass filters. Simultaneous and parfocal capture of video sequences were recorded for both wavelengths, with each wavelength on separate halves of the camera chip. Video recordings were made at 16 bit depth 2048 × 2048 resolution using a 10× objective (NA 0.40, Olympus, Tokyo, Japan) using an Orca Flash 4.0 v3 scientific digital camera (Hamamatsu, Hamamatsu City, Japan) and controlled by HCImage Live software (Hamamatsu, Hamamatsu City, Japan) on a desktop computer.

### Experimental protocol

To evaluate O_2_ and CO_2_ dependence on blood flow in the microcirculation both independently and simultaneously, a series of gas perturbations were imposed on the surface of the EDL muscle in contact with the gas exchange chamber membrane similar to those described previously (Sové et al., 2021). The series of 9 perturbations were conducted on multiple fields of view across the muscle at a focal depth within ∼60 µm of the surface. Each field was recorded during the following four O_2_ perturbations: 1) 1 min 7% O_2_ followed by 2 min 12% O_2_, 2) 1 min 7% O_2_ followed by 2 min 2% O_2_, 3) 1 min 12% O_2_ followed by 2 min of 7% O_2_, 4) 1 min 2% O_2_ followed by 2 min of 7% O_2_. CO_2_ was maintained at 5% throughout the O_2_ challenges, N_2_ made up the balance of the gasses delivered *via* the exchange chamber. Following completion of O_2_ challenges, recordings were made during the following four CO_2_ challenges: 5) 1 min of 5% CO_2_ then 2 min of 10% CO_2_, 6) 1 min of 5% CO_2_ then 2 min of 0% CO_2_, 7) 1 min of 10% CO_2_ then 2 min of 5% CO_2_, and 8) 1 min of 0% CO_2_ then 2 min of 5% CO_2_. [O_2_] was maintained at 7% throughout the duration of the CO_2_ challenges. The combined perturbation was as follows: 1 min of 7% O_2_ and 5% CO_2_, then 1 min of 2% O_2_ and 5% CO_2_, and lastly 2 min of 2% O_2_ and 10% CO_2_ with N_2_ being the balance of gasses delivered. Prior to the start of each perturbation the muscle was allowed to equilibrate for 1–2 min at the baseline O_2_ and CO_2_ concentrations for the next perturbation. Following the full series of 9 challenges the muscle was allowed to re-equilibrate at 7% [O_2_] and 5% [CO_2_] for 10 min prior to repeating the sequence in the next field of view.

Gas perturbations were imposed on the surface of the muscle using a three-dimensionally (3D) printed gas exchange chamber as described previously (Sové et al., 2021). The device in the present study employed an exchange window (5 mm × 3.5 mm) fitted with a 50 µm thick gas permeable membrane fabricated by spin coating polydimethylsiloxane onto a standard glass slide. The assembled device was connected by plastic tubing to a triple-inlet manifold supplied by three computer-controlled mass flow meters (SmartTrak100, Sierra Instruments, Monterey, CA, United States) for each gas channel, with a frequency response of <300 ms. The EDL muscle was placed over the exchange window of the device and isolated from room air as described above. Gas concentrations from the mass flow meters were dynamically controlled by custom MATLAB software allowing changes in O_2_ and CO_2_ concentrations to be triggered automatically at the appropriate time and sequence.

### Offline analysis and statistics

Offline analysis was conducted using custom software written in MATLAB (Mathworks, Natick, MA, United States). The software generated mp4 videos of captured sequences and functional images including variance and sum of absolute difference images used to facilitate identification of in-focus vessel segments for analysis (Japee et al., 2004). In focus capillaries with single file RBC flow were semi-automatically selected for analysis and space time images (STIs) were generated at each wavelength as described previously (Ellis et al., 1990, 1992, 2002, 2012; Japee et al., 2004; Ghonaim et al., 2011; Fraser et al., 2012). STIs were analyzed using the custom software package written in MATLAB and 1 second means were calculated from frame-by-frame measurements of velocity, lineal density, RBC supply rate (SR), and RBC oxygen saturation (SO_2_). Resulting second-by-second means were used for statistical comparisons.

Statistical analysis of the resulting capillary hemodynamic data was completed using Prism (Graphpad, California, United States). Time constants and associated parameters were determined using a mono-exponential non-linear least-squared curve fitting implemented in Prism for O_2_, CO_2_, and combined O_2_ and CO_2_ challenges, similar to approaches used to quantify V̇O_2_ kinetics (Tschakovsky et al., 2006). O_2_ and CO_2_ responses were fit to the following mono-exponential model:

$$Y\left(t\right)=Y_{b}+Y_{0}\left(1-e^{-\frac{t-X_{0}}{\tau }}\right)$$

where *Y(t)* is the response over time, *Y*_*b*_ is the magnitude at baseline, *X*_*0*_ is the time delay, *Y*_*0*_ is the asymptotic amplitude of the response, and *τ* is the time constant representing the time to reach 63% of the full response. Combined O_2_ and CO_2_ responses were fit to a multi-exponential model as follows:

$$Y\left(t\right)=Y_{b}+Y_{1}\left(1-e^{-\frac{t-X_{1}}{\tau_{1}}}\right)+Y_{2}\left(1-e^{-\frac{t-X_{2}}{\tau_{2}}}\right)$$

where *Y(t)* is the response over time, *Y*_*b*_ is the magnitude at baseline, *X*_*1*_ is the time delay, *Y*_*1*_ is the amplitude of the response to O_2_, τ_1_ is the time constant for the O_2_ response, *Y*_*2*_ is the amplitude of the CO_2_ response, *X*_2_ is the time delay, and τ_2_ is the time constant for the CO_2_ response. Repeated measures one-way ANOVA (using a mixed effects model to account for missing values) and Dunnett’s multiple comparisons were used to evaluate differences in the baseline period and the second-by-second mean responses following gas changes in the 3- and 4-min challenges. A *p*-value of <0.05 was considered significant. Mean and standard deviation are reported unless otherwise noted.

## Results

### Systemic animal data

Animal weight and systemic physiological animal monitoring data are shown in Table 1. Animal weights were measured immediately prior to the experiment. Reported mean arterial, systolic, and diastolic blood pressures represent the values recorded from the start of the capturing protocol and therefore include periods immediately following administration of anaesthetic. Mechanical ventilation respiratory rate and stroke volume are reported in Table 1. Arterial blood gasses are listed in Table 1.

**TABLE 1:** Systemic physiological and arterial blood gas measurements.

	Mean ± standard deviation
**Age (days)**	42.2 ± 1.3
**Animal Weight (g)**	187.5 ± 12.1
**Mean Arterial Pressure (mmHg)**	94.6 ± 5.8
**Systolic Blood Pressure (mmHg)**	109.9 ± 7.1
**Diastolic Blood Pressure (mmHg)**	78.2 ± 5.4
**Heart Rate (beats/min)**	406.1 ± 23.4
**Respiratory Rate (breaths/min)**	82.85 ± 2.12
**pH**	7.4 ± 0.02
**PCO**_**2**_ **(mmHg)**	48.7 ± 3.8
**PO**_**2**_ **(mmHg)**	108.2 ± 10.6
**BEecf (mmol/L)**	6.3 ± 2.0
**HCO**_**3**_ **(mmol/L)**	31.0 ± 2.0
**TCO**_**2**_ **(mmol/L)**	32.5 ± 1.9
**SO**_**2**_ **(%)**	98.3 ± 0.9
**Lac (mmol/L)**	0.89 ± 0.4

PCO_2_: partial pressure of carbon dioxide; PO_2_: partial pressure of oxygen.

BEecf: base excess in the extracellular fluid compartment concentration; HCO_3_.

bicarbonate concentration; TCO_2_: total carbon dioxide; SO_2_: oxygen saturation; Lac:

lactate concentration. *n* = 13 animals.

### Oxygen challenges

On-transient O_2_ mediated flow responses were measured during the 1-min baseline period at 7% [O_2_] followed by 12% high and 2% low O_2_ challenges for the remaining 2 min with a constant 5% [CO_2_] throughout the sequence. Off-transient flow responses were measured over 1-min periods of 12% (high), and 2% (low) chamber [O_2_] followed by 2 min of baseline 7% [O_2_]. Modeled parameters determined by non-linear least-squared fitting to mono-exponential curves for hemodynamic and capillary RBC SO_2_ following oxygen perturbation curves are listed in Table 2, stable exponential fits were determined for each hemodynamic and saturation measure in response to the O_2_ challenges.

**TABLE 2:** Parameters and constraints for mono-exponential non-linear least squared fit modeling of oxygen challenge responses.

Measurement	O_2_ challenge (%)	Modeled parameters				Constraints	
		**X**_**0**_	**Y**_**0**_	τ	**R** ^**2**^	**X**_**0**_	**Ranges**
**Mean Capillary RBC Velocity (μm/s)**	7–12	66.7	−29.4	7.0	0.8139	>60	51–180
	12–7	60	49.5	38.5	0.8468	>60	51–180
	7–2	60	116.5	35.5	0.9165	>60	51–180
	2–7	61.7	−66.3	13.4	0.8634	**>59**	51–180
**Mean Capillary RBC Lineal Density (cells/mm)**	7–12	64.9	−13.9	24.0	0.906	>60	51–180
	12–7	64.6	12.1	35.9	0.9547	>60	51–180
	7–2	64.7	13.5	26.9	0.9415	>60	51–180
	2–7	64.7	−11.1	68.2	0.8729	>60	51–180
**Mean Capillary Hematocrit (%)**	7–12	65.7	−4.9	27.2	0.9372	>60	51–180
	12–7	64.7	4.4	35.2	0.9475	>60	51–180
	7–2	63.2	5.4	32.4	0.9582	>60	51–180
	2–7	63.0	−4.1	74.8	0.88	>60	51–180
**Mean Capillary RBC Supply Rate (cells/s)**	7–12	63.0	−4.3	20.6	0.9301	>60	51–180
	12–7	60	5.2	36.1	0.9378	>60	51–180
	7–2	60	10.5	41.9	0.9591	>60	51–180
	2–7	61.4	−6.0	23.1	0.8763	>60	51–180
**Mean Capillary RBC Saturation (%)**	7–12	60.6	18.7	1.0	0.9923	>60	**0–90**
	12–7	62.2	−18.4	1.6	0.9909	>60	**NSV**
	7–2	62.3	−27.8	1.4	0.9952	>60	**51–90**
	2–7	62.7	24.5	1.3	0.9929	>60	**NSV**

Bolded values represent constraints set for fitting individual responses that diverge from the general approach applied to model responses. Constraints were required to obtain a stable non-linear least-squared fit for the sequences indicated. NSV: no set value.

**FIGURE 1:**
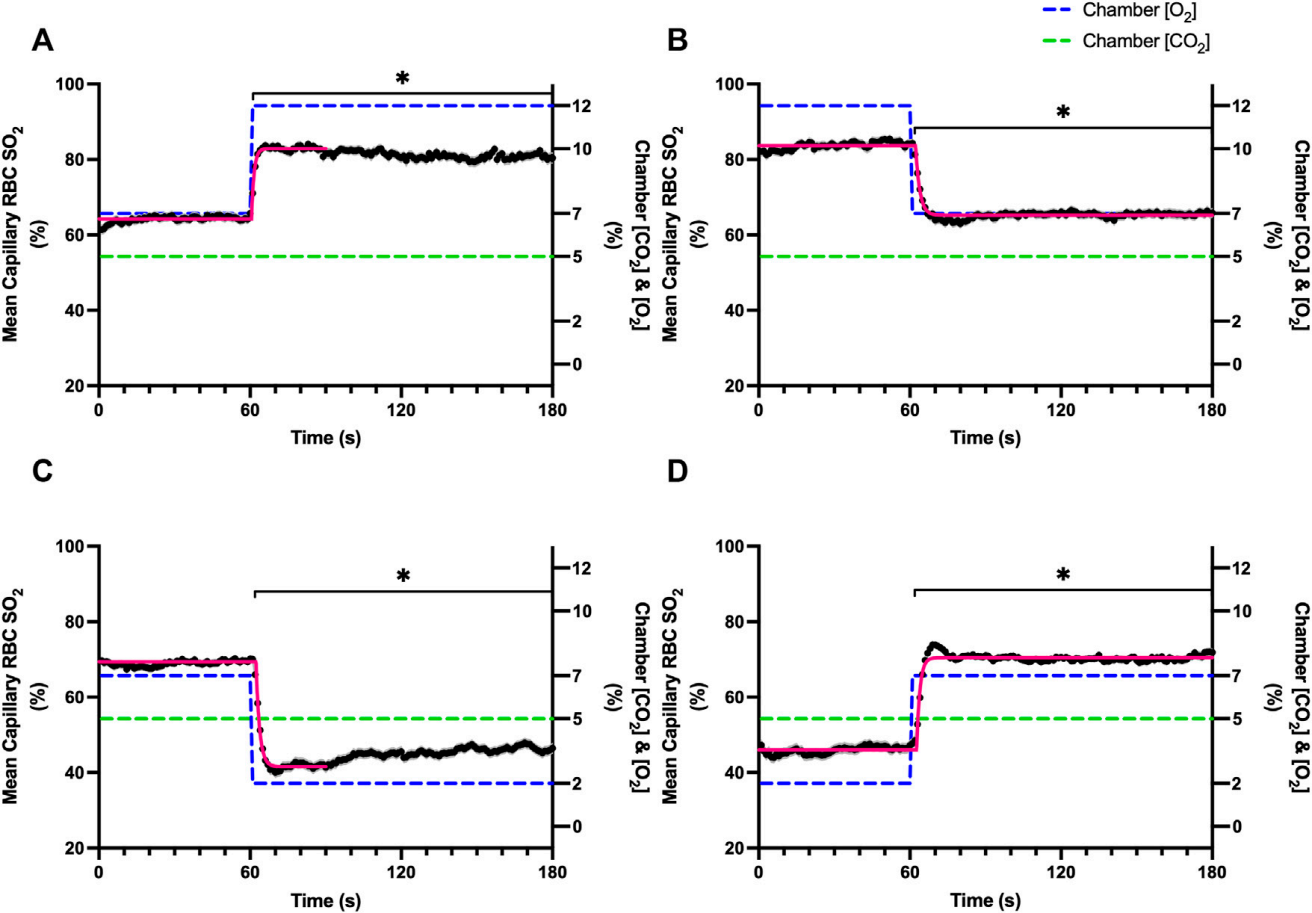
Mean capillary red blood cell oxygen saturation in response to O_2_ challenges. Second-by-second capillary red blood cell (RBC) oxygen saturation (SO_2_) measurements were made from intravital video microscopy of rat skeletal muscle microcirculation, recorded during four different stepwise O_2_ challenges. Each oxygen challenge began with a 1-min baseline period where chamber O_2_ concentration ([O_2_]) was set to a low (2%), normal (7%), or high (12%) concentration as the initial [O_2_]. Following the baseline period, the chamber [O_2_] was abruptly changed to the concentration of interest for the remaining 2-min so that hemodynamic responses could be quantified. The regime of [O_2_] challenges were 7–12% **(A)**, 12–7% **(B)**, 7–2% **(C)**, and 2–7% **(D)** with a steady 5% [CO_2_] and the balance of the gas mixture being composed of N_2_. The mean SO_2_ at each time point consists of all capillaries measured during a given challenge, with the shaded region representing the standard error of the mean. Resulting mean responses were modeled to a mono-exponential using established non-linear least squared fitting methods to aid in describing the dynamics of responses. Time constants (*τ*) for each SO_2_ response were 1.01 s (Panel A, *n* = 167), 1.44 s (Panel B, *n* = 168), 1.64 s (Panel C, *n* = 221) and 1.27 s (Panel D, *n* = 214). A repeated measures one-way ANOVA (using a mixed effects model to account for missing values) was used to compare the last 10 s of the baseline period to the second-by-second mean responses following the step-change, the Dunnett’s multiple comparisons test was used. *p* < 0.05 (*) were considered to be significant.

**FIGURE 2:**
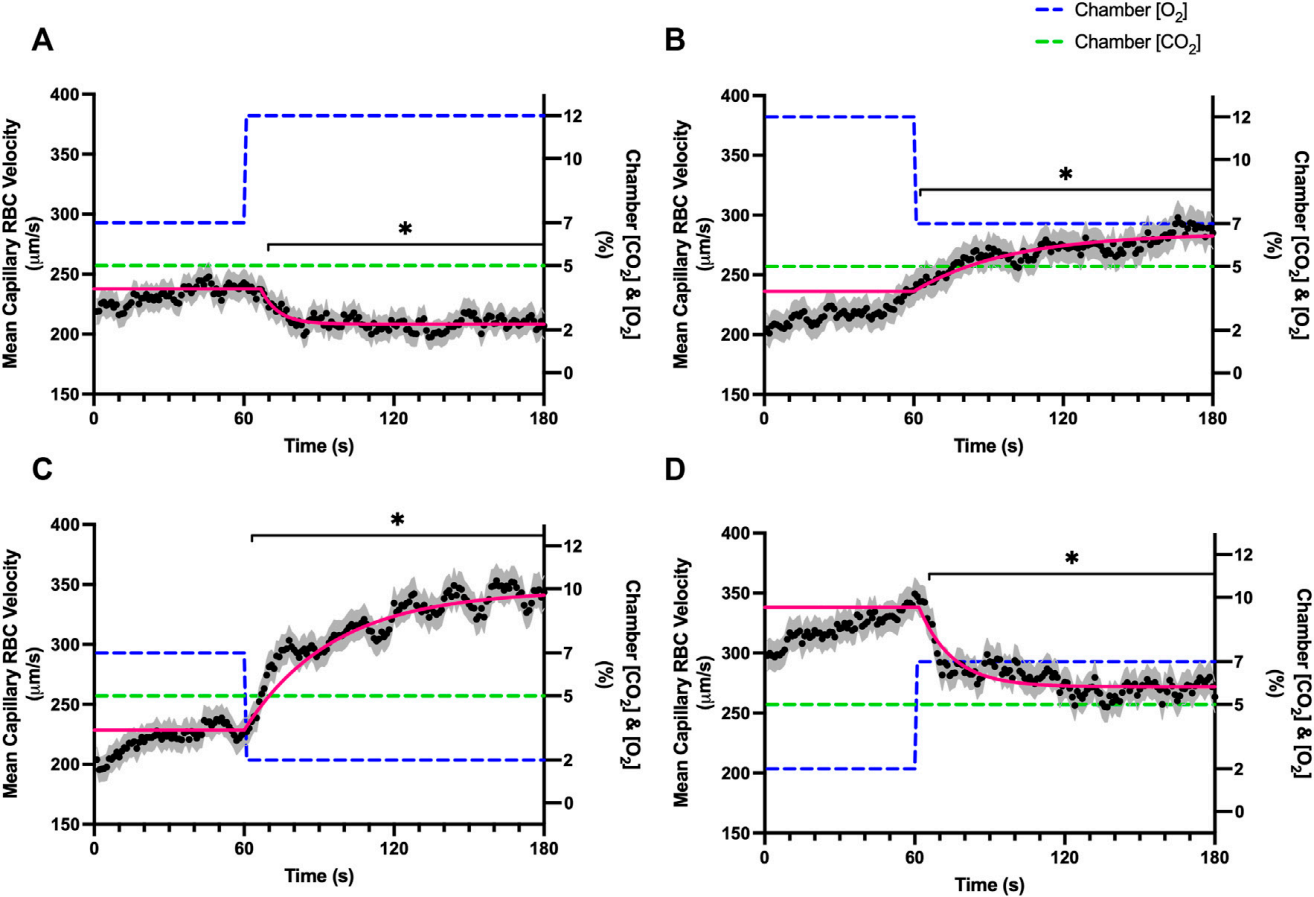
Mean capillary red blood cell velocity in response to O_2_ challenges. Second-by-second capillary red blood cell (RBC) velocity measurements were made from intravital video microscopy of rat skeletal muscle microcirculation, recorded during four different stepwise O_2_ challenges. Each oxygen challenge began with a 1-min baseline period where chamber O_2_ concentration ([O_2_]) was set to a low (2%), normal (7%), or high (12%) concentration as the initial [O_2_]. Following the baseline period, the chamber [O_2_] was abruptly changed to the concentration of interest for the remaining 2-min so that hemodynamic responses could be quantified. The regime of [O_2_] challenges were 7–12% **(A)**, 12–7% **(B)**, 7–2% **(C)**, and 2–7% **(D)** with a steady 5% [CO_2_] and the balance of the gas mixture being composed of N_2_. The mean velocity at each time point consists of all capillaries measured for a given challenge, with the shaded region representing the standard error of the mean. Resulting mean responses were modeled to a mono-exponential using established non-linear least squared fitting methods to aid in describing the dynamics of responses. Time constants (*τ*) for each velocity response were 6.96 s (Panel A, n = 272 capillaries), 35.54 s (Panel B, n = 294), 38.54 s (Panel C, n = 300) and 13.36 s (Panel D, n = 293). A repeated measures one-way ANOVA (using a mixed effects model to account for missing values) was used to compare the last 10s of the baseline period to the second-by-second mean responses following the step-change, the Dunnett’s multiple comparisons test was used. *p* < 0.05 (*) were considered to be significant.

**FIGURE 3:**
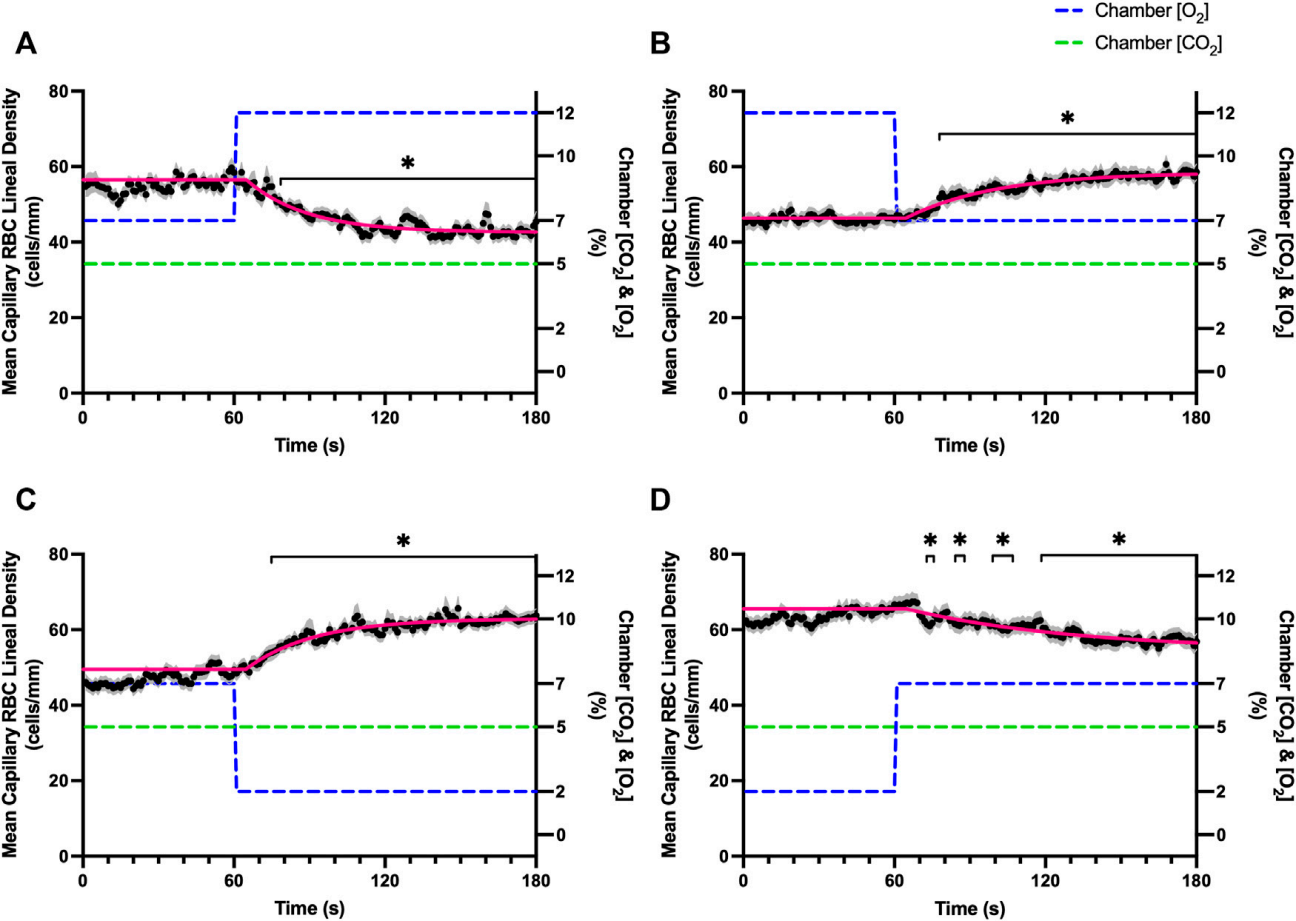
Mean capillary red blood cell lineal density in response to O_2_ challenges. Second-by-second capillary red blood cell (RBC) lineal density (LD) measurements were made from intravital video microscopy of rat skeletal muscle microcirculation, recorded during four different stepwise O_2_ challenges. Each oxygen challenge began with a 1-min baseline period where chamber O_2_ concentration ([O_2_]) was set to a low (2%), normal (7%), or high (12%) concentration as the initial [O_2_]. Following the baseline period, the chamber [O_2_] was abruptly changed to the concentration of interest for the remaining 2-min so that hemodynamic responses could be quantified. The regime of [O_2_] challenges were 7–12% **(A)**, 12–7% **(B)**, 7–2% **(C)**, and 2–7% **(D)** with a steady 5% [CO_2_] and the balance of the gas mixture being composed of N_2_. The mean LD at each time point consists of all capillaries measured during a given challenge, with the shaded region representing the standard error of the mean. Resulting mean responses were modeled to a mono-exponential using established non-linear least squared fitting methods to aid in describing the dynamics of responses. Time constants (*τ*) for each LD response were 24.03 s (Panel A, *n* = 279), 26.93 s (Panel B, *n* = 298), 35.93 s (Panel C, *n* = 305) and 68.24 s (Panel D, *n* = 294). A repeated measures one-way ANOVA (using a mixed effects model to account for missing values) was used to compare the last 10 s of the baseline period to the second-by-second mean responses following the step-change, the Dunnett’s multiple comparisons test was used. *p* < 0.05 (*) were considered to be significant.

**FIGURE 4:**
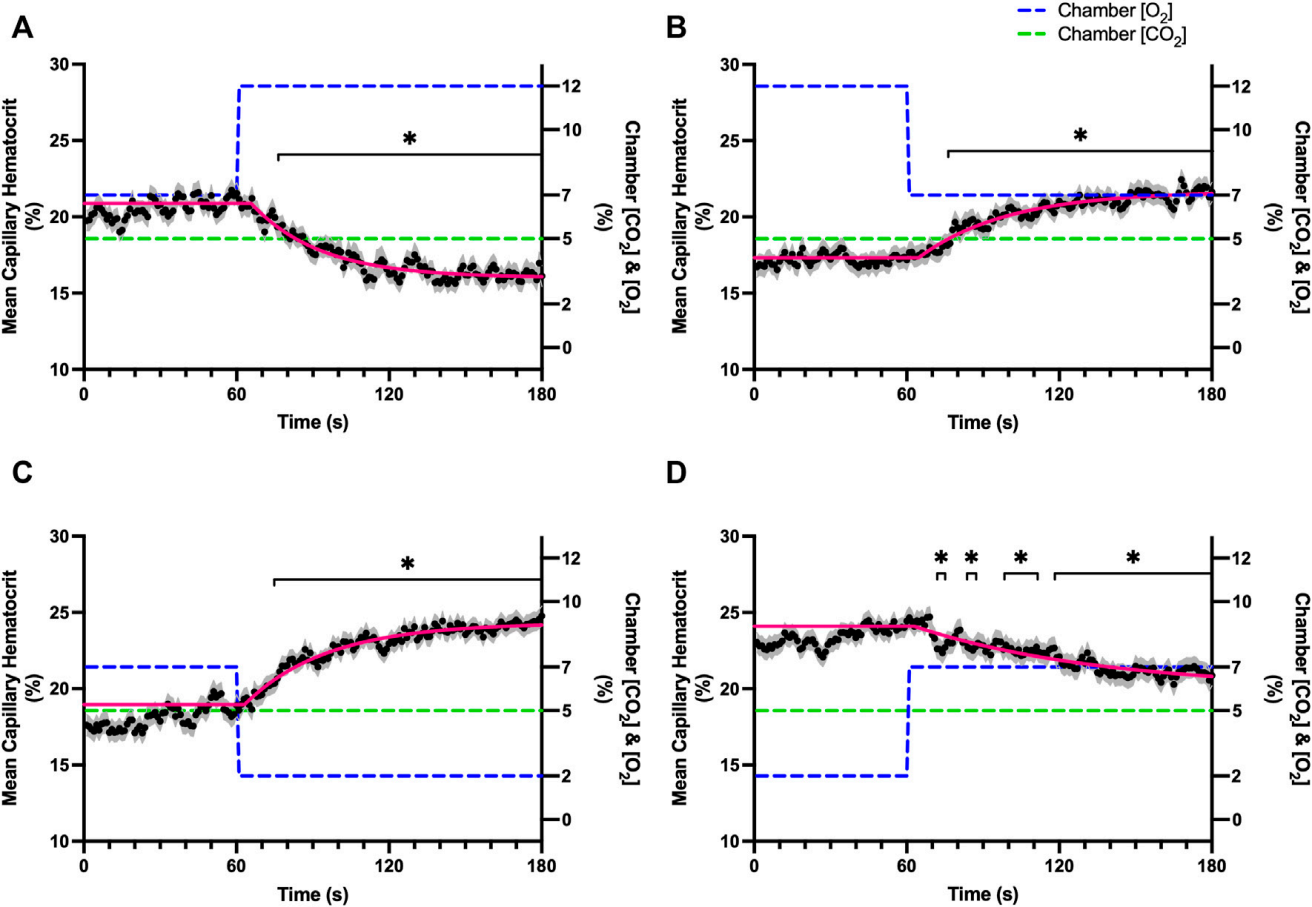
Mean capillary red blood cell hematocrit in response to O_2_ challenges. Second-by-second capillary red blood cell (RBC) hematocrit measurements were made from intravital video microscopy of rat skeletal muscle microcirculation, recorded during four different stepwise O_2_ challenges. Each oxygen challenge began with a 1-min baseline period where chamber O_2_ concentration ([O_2_]) was set to a low (2%), normal (7%), or high (12%) concentration as the initial [O_2_]. Following the baseline period, the chamber [O_2_] was abruptly changed to the concentration of interest for the remaining 2-min so that hemodynamic responses could be quantified. The regime of [O_2_] challenges were 7–12% **(A)**, 12–7% **(B)**, 7–2% **(C)**, and 2–7% **(D)** with a steady 5% [CO_2_] and the balance of the gas mixture being composed of N_2_. The mean hematocrit at each time point consists of all capillaries measured during a given challenge, with the shaded region representing the standard error of the mean. Resulting mean responses were modeled to a mono-exponential using established non-linear least squared fitting methods to aid in describing the dynamics of responses. Time constants (*τ*) for each hematocrit response were 27.19 s (Panel A, *n* = 278), 32.42 s (Panel B, *n* = 298), 35.21 s (Panel C, *n* = 305) and 74.75 s (Panel D, *n* = 294). A repeated measures one-way ANOVA (using a mixed effects model to account for missing values) was used to compare the last 10 s of the baseline period to the second-by-second mean responses following the step-change, the Dunnett’s multiple comparisons test was used. *p* < 0.05 (*) were considered to be significant.

**FIGURE 5:**
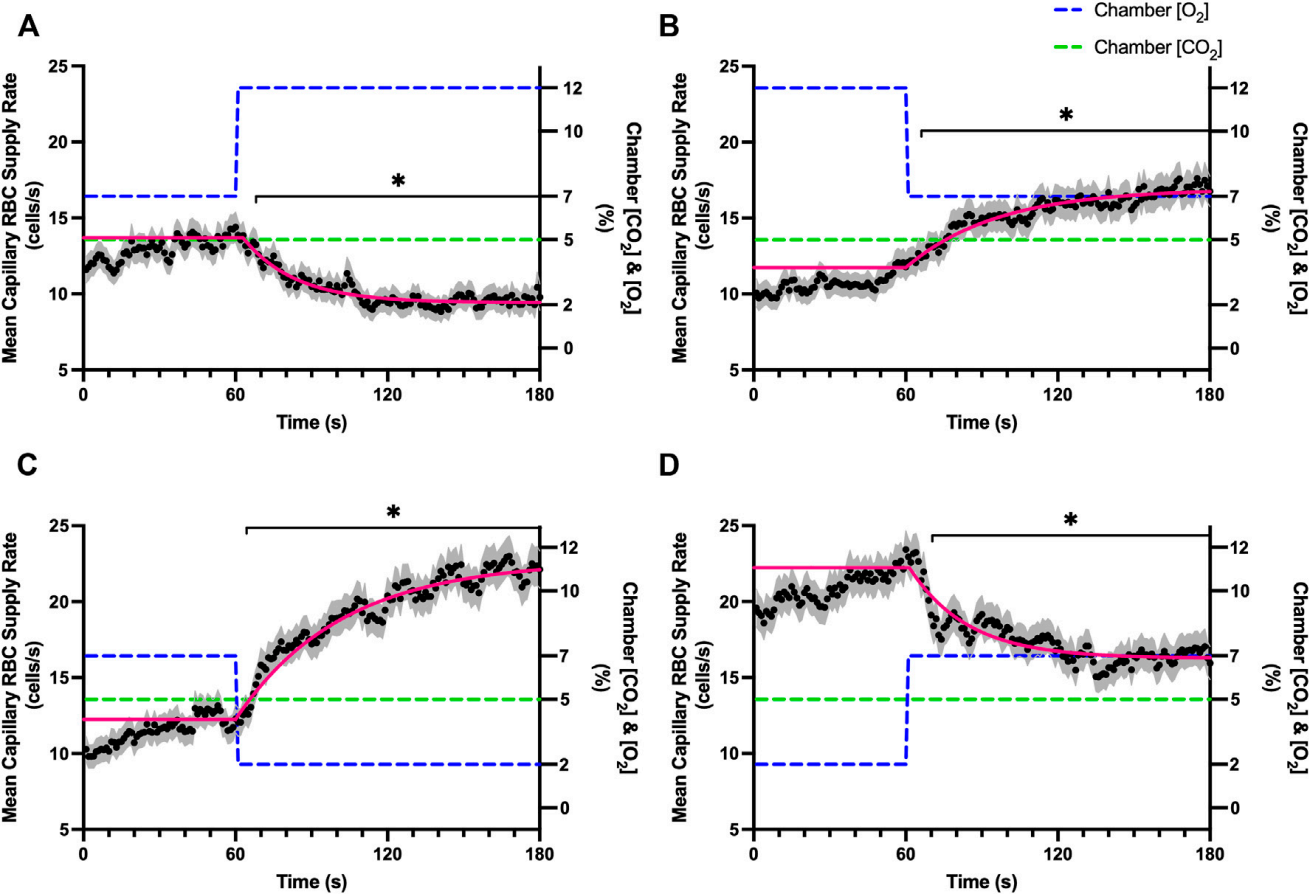
Mean capillary red blood cell supply rate in response to O_2_ challenges. Second-by-second capillary red blood cell (RBC) supply rate (SR) measurements were made from intravital video microscopy of rat skeletal muscle microcirculation, recorded during four different stepwise O_2_ challenges. Each oxygen challenge began with a 1-min baseline period where chamber O_2_ concentration ([O_2_]) was set to a low (2%), normal (7%), or high (12%) concentration as the initial [O_2_]. Following the baseline period, the chamber [O_2_] was abruptly changed to the concentration of interest for the remaining 2-min so that hemodynamic responses could be quantified. The regime of [O_2_] challenges were 7–12% **(A)**, 12–7% **(B)**, 7–2% **(C)**, and 2–7% **(D)** with a steady 5% [CO_2_] and the balance of the gas mixture being composed of N_2_. The mean SR at each time point consists of all capillaries measured during a given challenge, with the shaded region representing the standard error of the mean. Resulting mean responses were modeled to a mono-exponential using established non-linear least squared fitting methods to aid in describing the dynamics of responses. Time constants (*τ*) for each SR response were 20.61 s (Panel A, n = 278), 41.87 s (Panel B, n = 298), 36.06 s (Panel C, n = 304) and 23.07 s (Panel D, n = 294). A repeated measures one-way ANOVA (using a mixed effects model to account for missing values) was used to compare the last 10s of the baseline period to the second-by-second mean responses following the step-change, the Dunnett’s multiple comparisons test was used. *p* < 0.05 (*) were considered to be significant.

Increases in [O_2_] from 2–7% and 7–12% caused significant increases in RBC SO_2_ within 2 s of the step change, compared to the respective baseline periods (Table 3; Figures 1A,D). Significant decreases in RBC velocity, lineal density, capillary hematocrit, and RBC supply rate, were observed in response to increased [O_2_] (Table 3; Figures 2–5). Capillary RBC velocity decreased by 66 s following the 2–7% change in [O_2_]; whereas lineal density did not show significantly decreases until 79 s (Table 3; Figures 2–5). Decreasing [O_2_] from 12 to 7% and 7 to 2% resulted in significant decreases in RBC SO_2_ by 62 s after the step change in [O_2_] (Table 3; Figures 1B,C). Significant increases in all measured hemodynamic parameters were observed within 18 s following the decrease in [O_2_] (Table 3; Figures 2–5). Changes in RBC SO_2_ in response to increased [O_2_] from 7 to 12%, yielded the fastest time transient (*τ* = 1.009 s), while the slowest time constant was observed with capillary hematocrit in response to increases in [O_2_] from 2—7% (*τ* = 74.75 s). Time constants were fastest for saturation changes and slowest for lineal density and capillary hematocrit responses.

**TABLE 3:** Mean capillary blood flow responses to oxygen challenges.

	O_2_ challenge (%)	Mean of baseline period (51–60 s)	First significant mean response post challenge (61–180 s)	Time of first significant mean response post challenge (s)	*p*-value of first significant mean response	Peak/nadir of mean response post challenge	Time of peak/nadir of mean response post challenge (s)	*p*-value of peak/nadir response
Saturation (%)	7–12	64.4 ± 14.7	65.7 ± 15.2	61	0.0026	84.1 ± 12.7	84	<0.0001
	12–7	84.5 ± 13.9	81.3 ± 13.5	62	<0.0001	63.0 ± 15.2	80	<0.0001
	7–2	69.7 ± 13.1	65.9 ± 13.9	62	<0.0001	40.1 ± 19.7	70	<0.0001
	2–7	46.5 ± 19.6	48.5 ± 19.0	62	0.0001	73.9 ± 13.2	70	<0.0001
Velocity (µm/s)	7–12	238.6 ± 183.4	222.3 ± 175.6	70	0.0051	197.5 ± 155.7	134	<0.0001
	12–7	230.6 ± 206.6	242.0 ± 211.7	61	<0.0001	297.9 ± 238.6	166	<0.0001
	7–2	228.5 ± 190.4	235.7 ± 193.5	63	0.0003	353.2 ± 238.6	161	<0.0001
	2–7	337.2 ± 221.5	323.6 ± 214.7	66	0.0054	254.9 ± 200.6	137	<0.0001
Lineal Density (cells/mm)	7–12	56.5 ± 36.6	49.9 ± 30.3	79	0.0282	41.2 ± 29.3	143	<0.0001
	12–7	46.3 ± 34.0	51.8 ± 38.4	78	0.0006	60.7 ± 38.1	168	<0.0001
	7–2	49.4 ± 34.2	54.3 ± 33.1	76	0.0105	65.6 ± 53.0	149	<0.0001
	2–7	65.1 ± 35.3	61.4 ± 38.1	73	0.0342	55.0 ± 35.6	165	<0.0001
Hematocrit (%)	7–12	20.9 ± 10.9	19.3 ± 11.2	77	0.0278	15.6 ± 10.2	143	<0.0001
	12–7	17.2 ± 11.2	19.2 ± 12.3	78	0.0016	22.4 ± 11.2	168	<0.0001
	7–2	19.0 ± 12.2	20.7 ± 10.7	76	0.0038	24.8 ± 11.2	180	<0.0001
	2–7	24.0 ± 10.7	22.6 ± 11.6	73	0.0098	20.3 ± 11.8	165	<0.0001
Supply Rate (cells/s)	7–12	13.7 ± 15.3	12.6 ± 14.8	69	0.0432	8.8 ± 11.8	141	<0.0001
	12–7	11.5 ± 14.9	13.1 ± 16.6	69	0.0003	17.6 ± 18.8	175	<0.0001
	7–2	12.3 ± 15.9	13.9 ± 16.3	67	0.0194	23.0 ± 23.2	168	<0.0001
	2–7	22.2 ± 20.6	20.8 ± 20.2	68	0.0018	15.0 ± 17.5	136	<0.0001

### Carbon dioxide challenges

On-transient CO_2_ mediated flow responses were measured during the 1-min baseline period at 5% followed by 0% low and 10% high [CO_2_] challenge for the remaining 2 min with stable 7% [O_2_] throughout. Off-transient challenges were measured by 1-min periods of 0% low and 10% high CO_2_ with sequential change to 5% CO_2_. Parameters defined for mono-exponential non-linear least squared fitting of CO_2_ perturbation curves are listed in Table 4.

**TABLE 4:** Parameters and constraints for mono-exponential non-linear least squared fit modeling of carbon dioxide challenge responses.

Measurement	CO_2_ challenge (%)	Modeled parameters				Constraints	
		**X**_**0**_	**Y**_**0**_	τ	**R** ^**2**^	**X**_**0**_	**Ranges**
**Mean Capillary RBC Velocity (um/s)**	5–0	60.7	−257.5	18.88	0.9902	>60	51–180
	0–5	66.5	275.8	21.79	0.9951	>60	51–180
	5–10	60	182.2	79.34	0.9735	>60	51–180
	10–5	62.8	−94.3	20.66	0.9686	>60	51–180
**Mean Capillary RBC Lineal Density (cells/mm)**	5–0	63.7	−41.8	15.89	0.9915	>60	**NSV**
	0–5	70.8	41.6	30.63	0.9929	**NSV**	**NSV**
	5–10	60	10.3	30.31	0.8855	>60	**NSV**
	10–5	-	-	-	-	-	-
**Mean Capillary Hematocrit (%)**	5–0	63.7	−13.7	15.3	0.9912	>60	51–180
	0–5	70.9	15.9	38.5	0.9920	>60	51–180
	5–10	60	4.0	65.9	0.8757	>60	51–180
	10–5	-	-	-	-	-	-
**Mean Capillary RBC Supply Rate (cells/s)**	5–0	61.6	−21.6	13.8	0.9966	>60	51–180
	0–5	63.0	17.1	33.0	0.9593	>60	51–180
	5–10	60	14.4	88.1	0.9962	>60	51–180
	10–5	65.9	−5.8	20.2	0.9479	>60	51–180
**Mean Capillary RBC Saturation (%)**	5–0	62.4	6.3	0.84	0.8509	>60	**51–100**
	0–5	67.8	−5.9	0.39	0.5907	>60	**51–100**
	5–10	60	−5.7	2.9	0.9026	>60	**51–100**
	10–5	60	6.1	5.4	0.8792	**= 60**	**51–180**

Bolded values represent constraints set for fitting individual responses that diverge from the general approach applied to model responses. Constraints were required to obtain a stable non-linear least-squared fit for the sequences indicated. No stable exponential fit was found for both the lineal density and hematocrit curves in response to the 10–5% [CO_2_] challenge. NSV: no set value.

**FIGURE 6:**
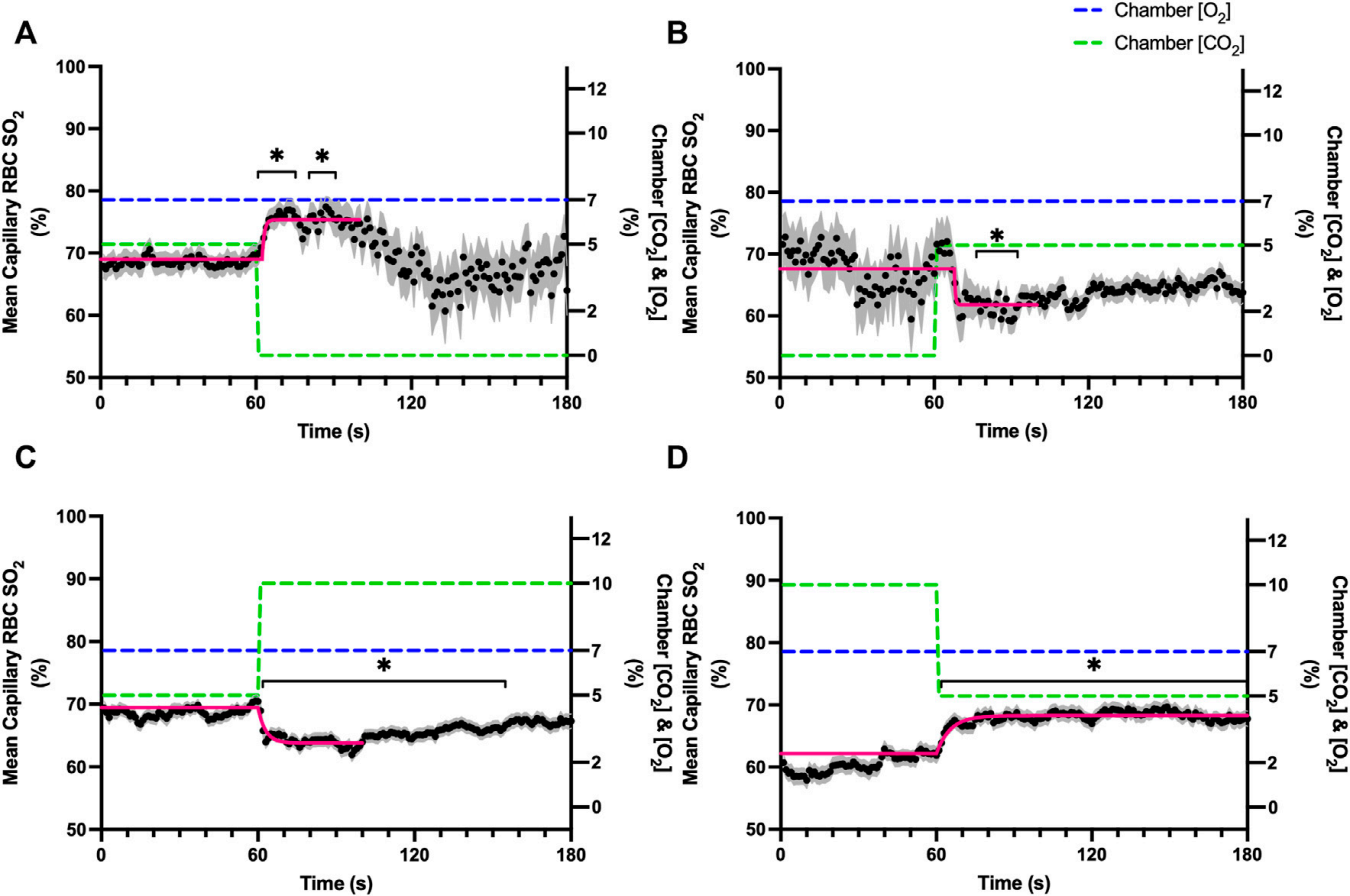
Mean capillary red blood cell oxygen saturation in response to CO_2_ challenges. Second-by-second capillary red blood cell (RBC) oxygen saturation (SO_2_) measurements were made from intravital video microscopy of rat skeletal muscle microcirculation, recorded during four different stepwise CO_2_ challenges. Each CO_2_ challenge began with a 1-min baseline period where chamber CO_2_ concentration ([CO_2_]) was set to a low (0%), normal (5%), or high (10%) concentration as the initial [CO_2_]. Following the baseline period, the chamber [CO_2_] was abruptly changed to the concentration of interest for the remaining 2-min so that hemodynamic responses could be quantified. The regime of [CO_2_] challenges were 5–0% **(A)**, 0–5% **(B)**, 5–10% **(C)**, and 10–5% **(D)** with a steady 7% [O_2_] and the balance of the gas mixture being composed of N_2_. The mean SO_2_ at each time point consists of all capillaries measured during a given challenge, with the shaded region representing the standard error of the mean. Resulting mean responses were modeled to a mono-exponential using established non-linear least squared fitting methods to aid in describing the dynamics of responses. Time constants (*τ*) for each SO_2_ response were 0.84 s (Panel A, *n* = 82), 0.38 s (Panel B, *n* = 90), 2.887 s (Panel C, *n* = 197) and 5.339 s (Panel D, *n* = 183). A repeated measures one-way ANOVA (using a mixed effects model to account for missing values) was used to compare the last 10s of the baseline period to the second-by-second mean responses following the step-change, the Dunnett’s multiple comparisons test was used. *p* < 0.05 (*) were considered to be significant.

**FIGURE 7:**
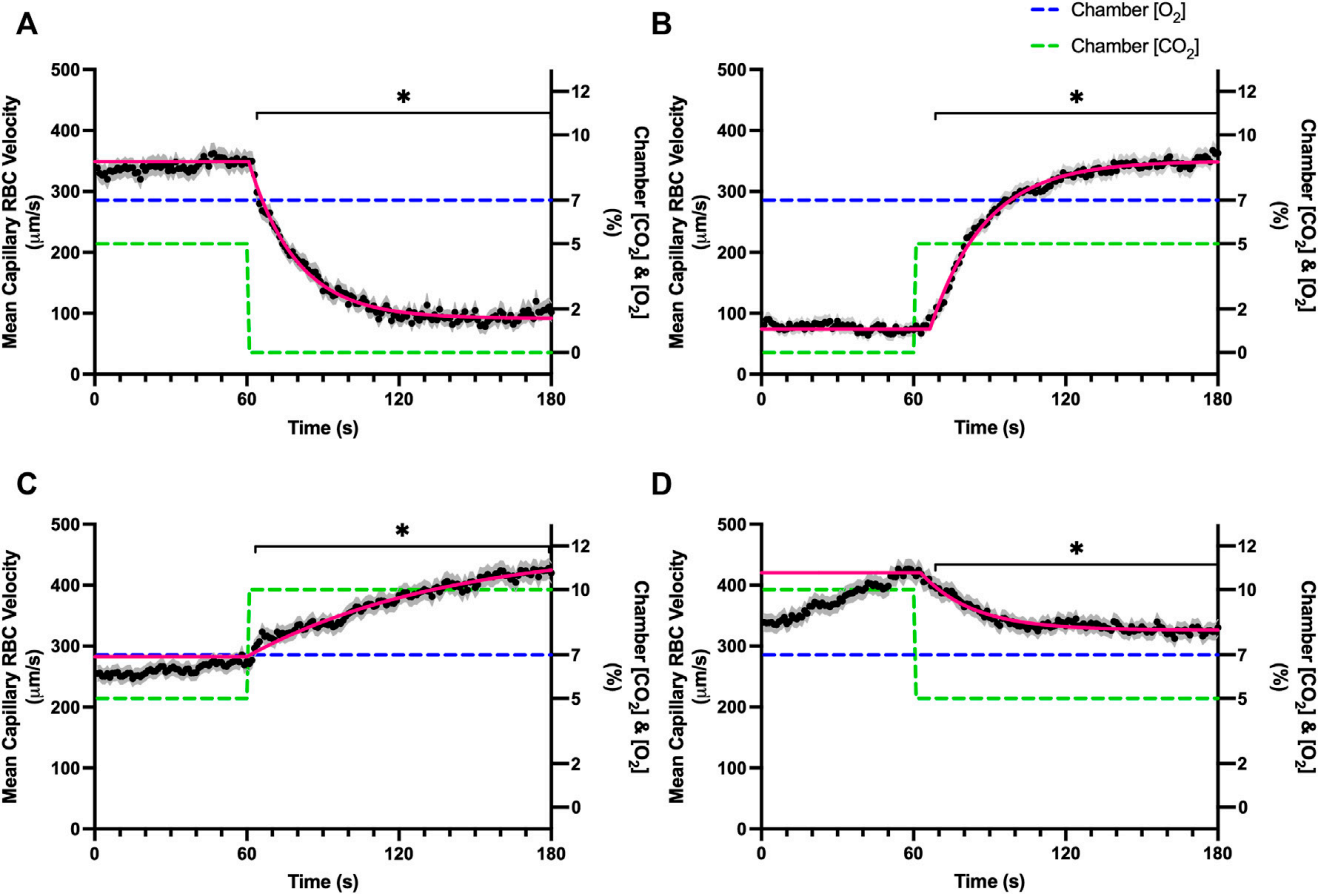
Mean capillary red blood cell velocity in response to CO_2_ challenges. Second-by-second capillary red blood cell (RBC) velocity measurements were made from intravital video microscopy of rat skeletal muscle microcirculation, recorded during four different stepwise CO_2_ challenges. Each CO_2_ challenge began with a 1-min baseline period where chamber CO_2_ concentration ([CO_2_]) was set to a low (0%), normal (5%), or high (10%) concentration as the initial [CO_2_]. Following the baseline period, the chamber [CO_2_] was abruptly changed to the concentration of interest for the remaining 2-min so that hemodynamic responses could be quantified. The regime of [CO_2_] challenges were 5–0% **(A)**, 0–5% **(B)**, 5–10% **(C)**, and 10–5% **(D)** with a steady 7% [O_2_] and the balance of the gas mixture being composed of N_2_. The mean velocity at each time point consists of all capillaries measured during a given challenge, with the shaded region representing the standard error of the mean. Resulting mean responses were modeled to a mono-exponential using established non-linear least squared fitting methods to aid in describing the dynamics of responses. Time constants (*τ*) for each velocity response were 18.88 s (Panel A, *n* = 245), 21.79 s (Panel B, *n* = 242), 79.34 s (Panel C, *n* = 288) and 20.66 s (Panel D, *n* = 285). A repeated measures one-way ANOVA (using a mixed effects model to account for missing values) was used to compare the last 10s of the baseline period to the second-by-second mean responses following the step-change, the Dunnett’s multiple comparisons test was used. *p* < 0.05 (*) were considered to be significant.

**FIGURE 8:**
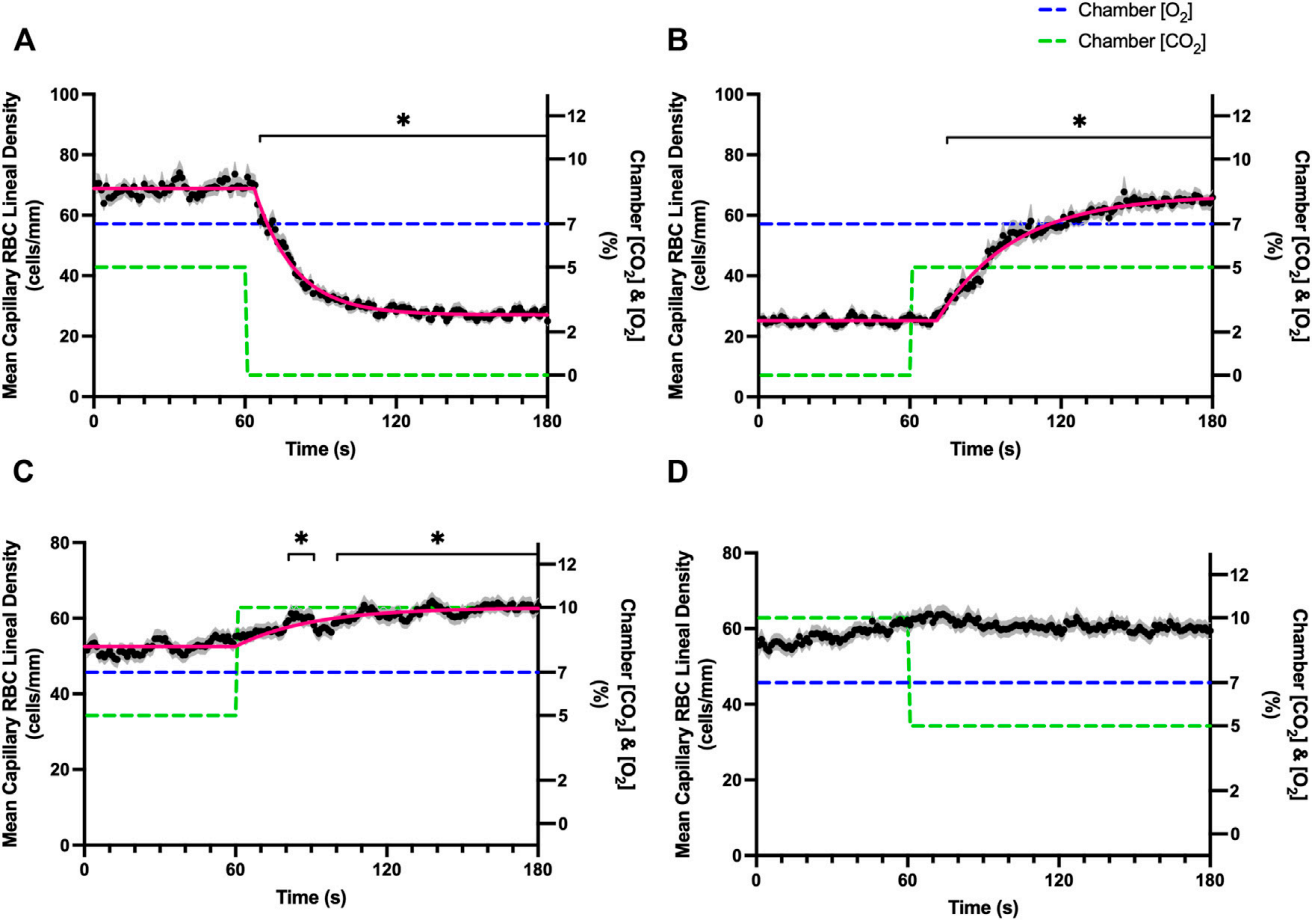
Mean capillary red blood cell lineal density in response to CO_2_ challenges. Second-by-second capillary red blood cell (RBC) lineal density (LD) measurements were made from intravital video microscopy of rat skeletal muscle microcirculation, recorded during four different stepwise CO_2_ challenges. Each CO_2_ challenge began with a 1-min baseline period where chamber CO_2_ concentration ([CO_2_]) was set to a low (0%), normal (5%), or high (10%) concentration as the initial ([CO_2_]). Following the baseline period, the chamber [CO_2_] was abruptly changed to the concentration of interest for the remaining 2-min so that hemodynamic responses could be quantified. The regime of [CO_2_] challenges were 5–0% **(A)**, 0–5% **(B)**, 5–10% **(C)**, and 10–5% **(D)** with a steady 7% [O_2_] and the balance of the gas mixture being composed of N_2_. The mean LD at each time point consists of all capillaries measured during a given challenge, with the shaded region representing the standard error of the mean. Resulting mean responses were modeled to a mono-exponential using established non-linear least squared fitting methods to aid in describing the dynamics of responses. Time constants (*τ*) for each LD response were 15.89 s (Panel A, *n* = 255), 30.63 s (Panel B, *n* = 246) and 30.31 s (Panel C, *n* = 292); there was no time constant for 10–5% (Panel D, *n* = 289). A repeated measures one-way ANOVA (using a mixed effects model to account for missing values) was used to compare the last 10 s of the baseline period to the second-by-second mean responses following the step-change, the Dunnett’s multiple comparisons test was used. *p* < 0.05 (*) were considered to be significant.

**FIGURE 9:**
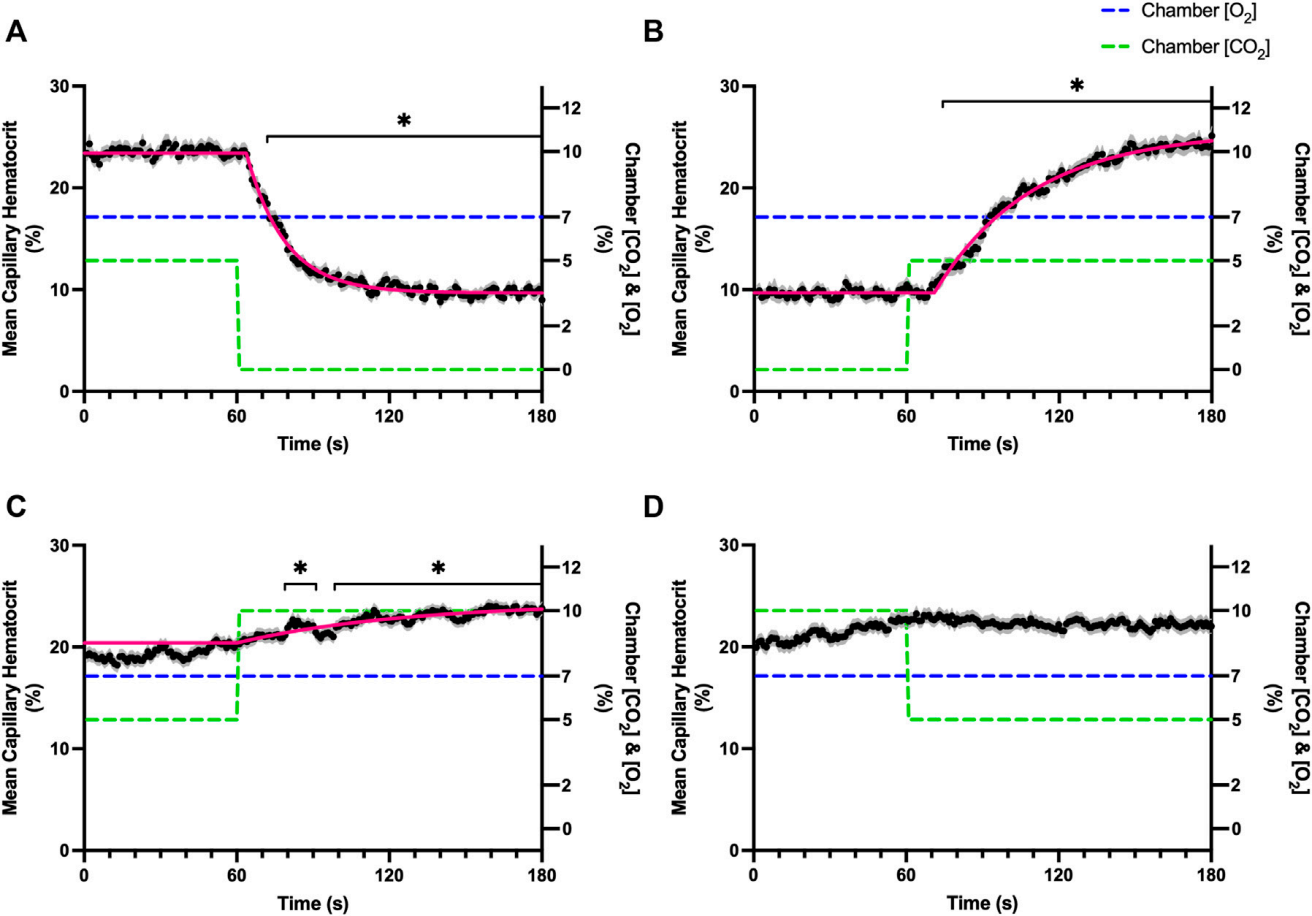
Mean capillary red blood cell hematocrit in response to CO_2_ challenges. Second-by-second capillary red blood cell (RBC) hematocrit measurements were made from intravital video microscopy of rat skeletal muscle microcirculation, recorded during four different stepwise CO_2_ challenges. Each CO_2_ challenge began with a 1-min baseline period where chamber CO_2_ concentration ([CO_2_]) was set to a low (0%), normal (5%), or high (10%) concentration as the initial ([CO_2_]). Following the baseline period, the chamber ([CO_2_]) was abruptly changed to the concentration of interest for the remaining 2-min so that hemodynamic responses could be quantified. The regime of ([CO_2_]) challenges were 5–0% **(A)**, 0–5% **(B)**, 5–10% **(C)**, and 10–5% **(D)** with a steady 7% [O_2_] and the balance of the gas mixture being composed of N_2_. The mean hematocrit at each time point consists of all capillaries measured during a given challenge, with the shaded region representing the standard error of the mean. Resulting mean responses were modeled to a mono-exponential using established non-linear least squared fitting methods to aid in describing the dynamics of responses. Time constants (*τ*) for each hematocrit response were 15.27 s (Panel A, *n* = 250), 38.50 s (Panel B, *n* = 248) and 65.94 s (Panel C, *n* = 292); there was no time constant for 10–5% ([CO_2_]) (Panel D, *n* = 289). A repeated measures one-way ANOVA (using a mixed effects model to account for missing values) was used to compare the last 10s of the baseline period to the second-by-second mean responses following the step-change, the Dunnett’s multiple comparisons test was used. *p* < 0.05 (*) were considered to be significant.

**FIGURE 10:**
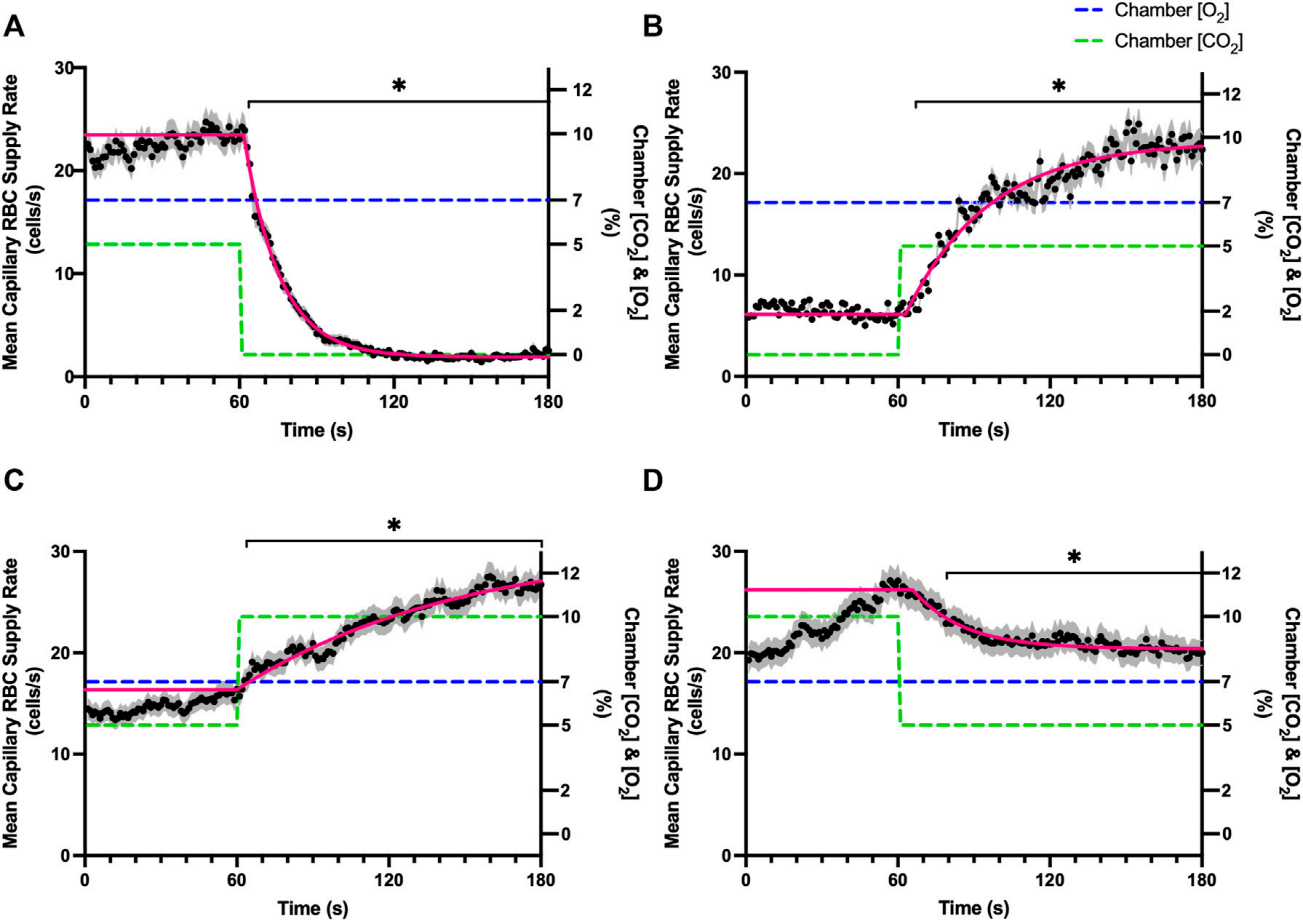
Mean capillary red blood cell supply rate in response to CO_2_ challenges. Second-by-second capillary red blood cell (RBC) supply rate (SR) measurements were made from intravital video microscopy of rat skeletal muscle microcirculation, recorded during four different stepwise CO_2_ challenges. Each CO_2_ challenge began with a 1-min baseline period where chamber CO_2_ concentration ([CO_2_]) was set to a low (0%), normal (5%), or high (10%) concentration as the initial ([CO_2_]). Following the baseline period, the chamber ([CO_2_]) was abruptly changed to the concentration of interest for the remaining 2-min so that hemodynamic responses could be quantified. The regime of ([CO_2_]) challenges were 5–0% **(A)**, 0–5% **(B)**, 5–10% **(C)**, and 10–5% **(D)** with a steady 7% [O_2_] and the balance of the gas mixture being composed of N_2_. The mean SR at each time point consists of all capillaries measured during a given challenge, with the shaded region representing the standard error of the mean. Resulting mean responses were modeled to a mono-exponential using established non-linear least squared fitting methods to aid in describing the dynamics of responses. Time constants (*τ*) for each SR response were 13.84 s (Panel A, *n* = 250), 32.96 s (Panel B, *n* = 246), 88.08 s (Panel C, *n* = 290) and 20.18 s (Panel D, *n* = 287). A repeated measures one-way ANOVA (using a mixed effects model to account for missing values) was used to compare the last 10 s of the baseline period to the second-by-second mean responses following the step-change, the Dunnett’s multiple comparisons test was used. *p* < 0.05 (*) were considered to be significant.

Reducing [CO_2_] from 5 to 0% and 10 to 5% lead to significant increases in RBC SO_2_ within 2 s compared to 51–60 s baseline period in both challenges (Table 5; Figures 6A,D). RBC velocity and RBC supply rate significantly decreased within 17 s of the step decrease in [CO_2_] (Figures 7A,D, 10A,D, respectively). Lineal density and capillary hematocrit were significantly decreased by 6 s after [CO_2_] was decreased from 5 to 0% (Figures 8A, 9A); however, lineal density and hematocrit did not significantly change in response to the 10–5% challenge (Figures 8D, 9D). Red blood cell SO_2_ significantly decreased in response to increased [CO_2_] both from 0 to 5% and 5–10% within 17 s following the change in [CO_2_] (Table 5; Figures 6B,C). There were significant increases in RBC velocity, lineal density, hematocrit, and RBC supply rate in response to increased [CO_2_] (Table 5; Figures 7–10). Increases in hemodynamic measurements occurred between 62 and 80 s following the change in gas conditions. The time constants for SO_2_ changes were among the fastest with tau ranging from 0.38 s to 5.3 s. The increased [CO_2_] from 5—10% resulted in the longest time constants for hemodynamic changes (*τ* = 30.3 s–88.1 s).

**TABLE 5:** Mean capillary blood flow responses to carbon dioxide challenges.

	CO_2_ challenge (%)	Mean of baseline period (51–60 s)	First significant mean response post challenge (61–180 s)	Time of first significant mean response post challenge (s)	*p*-value of first significant mean response	Peak/nadir of mean response post challenge	Time of peak/nadir of mean response post challenge (s)	*p*-value of peak/nadir response
Saturation (%)	5–0	68.7 ± 15.4	69.9 ± 14.4	61	0.0011	77.4 ± 13.3	87	0.0006
	0–5	65.7 ± 28.4	63.9 ± 16.9	77	0.0257	59.5 ± 19.3	90	0.0050
	5–10	69.5 ± 14.0	65.8 ± 14.2	62	<0.0001	61.9 ± 15.3	96	<0.0001
	10–5	62.1 ± 17.3	63.0 ± 19.7	62	<0.0001	69.7 ± 16.2	149	<0.0001
Velocity (µm/s)	5–0	349.1 ± 245.8	327.4 ± 235.4	63	<0.0001	78.2 ± 129.5	154	<0.0001
	0–5	71.4 ± 132.5	91.7 ± 162.3	67	0.0043	367.4 ± 249.0	178	<0.0001
	5–10	273.4 ± 218.1	276.8 ± 215.9	62	0.0007	427.6 ± 266.9	179	<0.0001
	10–5	420.4 ± 264.3	398.7 ± 266.3	67	0.0032	312.5 ± 222.8	163	<0.0001
Lineal Density (cells/mm)	5–0	70.3 ± 50.1	58.0 ± 36.5	66	<0.0001	25.0 ± 23.9	140	<0.0001
	0–5	25.0 ± 26.5	32.0 ± 34.1	75	0.0335	64.2 ± 34.4	180	<0.0001
	5–10	54.6 ± 35.0	58.6 ± 33.3	80	0.0003	64.5 ± 39.3	138	<0.0001
	10–5	60.8 ± 35.6	NA	NA	NA	NA	NA	NA
Hematocrit (%)	5–0	23.4 ± 12.0	20.8 ± 12.9	66	<0.0001	8.8 ± 8.1	140	<0.0001
	0–5	9.6 ± 10.2	12.3 ± 13.4	77	0.0397	25.1 ± 14.2	180	<0.0001
	5–10	22.4 ± 11.8	21.9 ± 10.9	80	0.0002	24.0 ± 11.0	159	<0.0001
	10–5	20.3 ± 11.3	NA	NA	NA	NA	NA	NA
Supply Rate (cells/s)	5–0	23.4 ± 22.4	20.6 ± 20.7	64	<0.0001	1.4 ± 4.2	154	<0.0001
	0–5	1.6 ± 6.0	2.7 ± 8.0	69	0.0035	23.7 ± 22.4	180	<0.0001
	5–10	15.9 ± 19.5	19.1 ± 20.8	66	<0.0001	27.5 ± 24.8	160	<0.0001
	10–5	26.3 ± 24.3	23.8 ± 22.3	77	0.0012	19.4 ± 20.7	174	<0.0001

### Combined O_2_ and CO_2_ challenges

Combined O_2_ and CO_2_ mediated flow responses were measured during a 4-min challenge. The 4-min sequence consisted of a 1-min baseline period with 7% [O_2_] and 5% [CO_2_], followed by a 1-min period with 2% [O_2_] and 5% [CO_2_], and lastly a 2-min period with 2% [O_2_] and 10% [CO_2_]. Parameters defined by mono-exponential non-linear least squared fitting hemodynamic and oxygen saturation responses to the combined O_2_ and CO_2_ perturbation are provided in Table 6. Stable exponential fits were achieved for all hemodynamic and saturation measures in response to the combined challenge.

**TABLE 6:** Parameters and constraints for multi-exponential non-linear least squared fit modeling of combined oxygen and carbon dioxide responses.

Measurement	Modelled parameters							Constraints		
	X_1_	Y_1_	X_2_	Y_2_	τ_1_	τ_2_	*R* ^2^	X_1_	X_2_	Ranges
**Mean Capillary RBC Velocity (μm/s)**	60	76.4	120	213.6	23.3	85.78	0.9843	>60	>120	51–240
**Mean Capillary RBC Lineal Density (cells/mm)**	64.4	8.0	120	3.2	22.38	23.63	0.9413	>60	>120	51–240
**Mean Capillary Hematocrit (%)**	64	2.9	120	1.4	21.52	20.73	0.9486	>60	>120	51–240
**Mean Capillary RBC Supply Rate (cells/s)**	62	6.6	120	11.3	20.94	49.87	0.9792	>60	>120	51–240
**Mean Capillary RBC Saturation (%)**	63.2	-20.9	120	15.9	0.6799	92.32	0.9561	>60	>120	51–240

**FIGURE 11:**
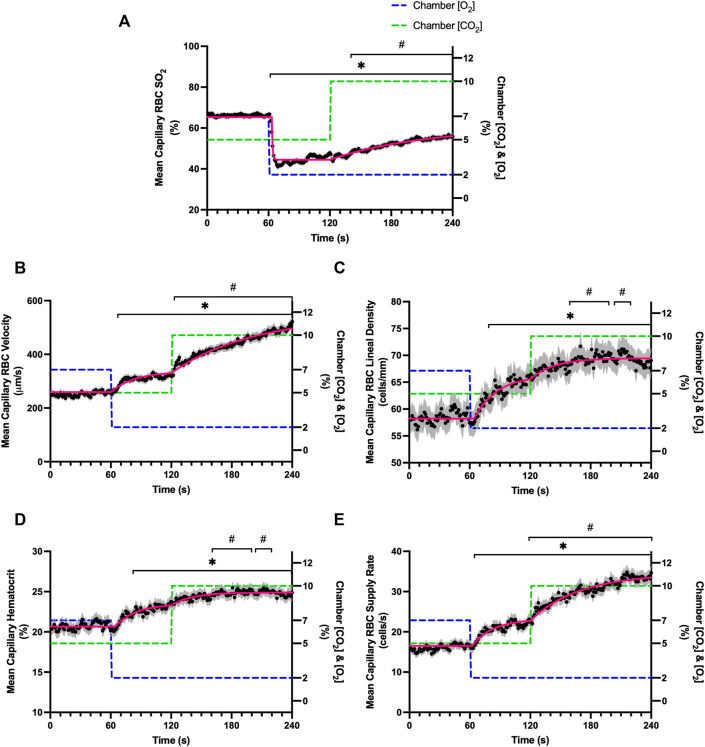
Mean hemodynamic measures in responses to combined O_2_ and CO_2_ challenges. Second-by-second capillary hemodynamic measurements were made from intravital video microscopy of rat skeletal muscle microcirculation, recorded during a 4-min combined O_2_ and CO_2_ stepwise challenge. For O_2_, the chamber O_2_ concentration ([O_2_]) was set to baseline 7% for 1-min, then decreased to 2% [O_2_] for the remaining 3 min. For CO_2_ concentrations ([CO_2_]), a 5% baseline period was set for 2 min, then [CO_2_] increased to 10% for the second 2 min period. During the 4-min sequence, the balance of the gas mixture was composed of N_2_. The hemodynamic parameters quantified were red blood cell (RBC) oxygen saturation (SO_2_) **(A)**, RBC velocity **(B)**, lineal density (LD) **(C)**, hematocrit **(D)**, and RBC supply rate (SR) **(E)**. Each time point consists of all capillaries measured during the combined challenge, with the shaded region representing the standard error of the mean. Resulting mean responses were modeled to a mono-exponential using established non-linear least squared fitting methods to aid in describing the dynamics of responses. Time constants (*τ*) for each O_2_ response were 23.30 s (Panel A, *n* = 286 capillaries), 22.38 s (Panel B, *n* = 292), 21.52 s (Panel C, *n* = 292), 20.94 s (Panel D, *n* = 291) and 0.68 s (Panel E, *n* = 193). Time constants (*τ*) for each CO_2_ response were 85.78 s (Panel A), 23.63 s (Panel B), 20.73 s (Panel C), 49.87 s (Panel D) and 92.32 s (Panel E). A repeated measures one-way ANOVA (using a mixed effects model to account for missing values) was used to compare the 51–60 s of the baseline period and the 111–120 s period to the second-by-second mean responses following the O_2_ and CO_2_ step-change, respectively, and the Dunnett’s multiple comparisons test was used. *: *p* < 0.05, compared to 51–60 s #: *p* < 0.05, compared to 111–120 s.

A significant decrease in RBC SO_2_ was observed by 62 s in response to a decrease in gas exchange chamber [O_2_] from 7–2%; however, this decrease in SO_2_ was followed by an increase in RBC SO_2_ at 142 s in response to increased [CO_2_] from 5–10% compared to the last 10 s of the low O_2_ period (111–120 s) (Table 7; Figure 11A). The combined challenge caused significant increases in RBC velocity by 67 s following the step change to 2% [O_2_] compared to the mean of the baseline period between 51–60 s, and subsequently velocity significantly increased by 123 s following the change from 5 to 10% [CO_2_] when compared to the low O_2_ period (111–120 s) (Figure 11B). The step change in gas exchange chamber [O_2_] from 7 to 2% caused significant increases in lineal density and mean capillary hematocrit by 83 s (Figures 11C,D respectively). The [CO_2_] step change from 5 to 10% during the combined challenge caused significant increases in lineal density by 162 s compared to the 111–120 s time period of the initial [O_2_] step change (Figure 11C). Similarly, hematocrit significantly increased by 161 s following the change to 10% [CO_2_] (Figure 11D). Mean capillary RBC SR significantly increased by 68 and 124 s in response to the combined step change in [O_2_] and [CO_2_] respectively (Figure 11E).

**TABLE 7:** Mean capillary blood flow responses to combined oxygen and carbon dioxide challenges.

	Mean of baseline (51–60 s)	First significant mean response post low O_2_ (61–120 s)	Time of first significant mean response post low O_2_ (s)	*p*-value of first significant mean response post low O_2_	Peak/nadir of mean response post low O_2_	Time of peak/nadir of mean response post low O_2_ (s)	*p*-value of peak/nadir response post low O_2_	Mean of low O_2_ (111–120 s)	First significant mean response post high CO_2_ (121–240 s)	Time of first significant mean response post high CO_2_ (s)	*p*-value of first significant mean response post high CO_2_	Peak/nadir of mean response post high CO_2_	Time of peak/nadir of mean response post high CO_2_ (s)	*p*-value of peak/nadir response post high CO_2_
Saturation (%)	66.1 ± 16.4	63.5 ± 16.6	62	0.0019	41.1 ± 18.9	69	<0.0001	46.2 ± 17.8	48.8 ± 17.9	142	0.0132	56.7 ± 19.6	236	<0.0001
Velocity (µm/s)	259.2 ± 194.2	276.8 ± 200.9	67	0.0031	331.5 ± 218.1	102	<0.0001	317.7 ± 209.3	344.9 ± 222.3	123	0.0106	521.3 ± 311.2	240	<0.0001
Lineal Density (cells/mm)	58.6 ± 39.9	62.8 ± 39.0	83	0.0123	66.4 ± 42.2	104	<0.0001	65.2 ± 38.6	69.0 ± 37.4	162	0.0114	71.7 ± 44.0	170	<0.0001
Hematocrit (%)	20.8 ± 13.1	22.3 ± 12.5	83	0.0107	23.8 ± 12.9	120	<0.0001	23.2 ± 12.5	24.6 ± 12.1	161	0.0295	25.5 ± 12.3	181	<0.0001
Supply Rate (cells/s)	16.6 ± 19.5	18.1 ± 20.1	68	0.0216	23.1 ± 22.7	119	<0.0001	22.0 ± 22.1	24.2 ± 22.9	124	0.0016	34.8 ± 30.4	216	<0.0001

## Discussion

The dynamics of V̇O_2_ uptake at the onset of exercise is coupled to the increased oxidative requirements of working skeletal muscle and the resulting time course of blood flow responses necessary to match oxygen demand (Andersen & Saltin, 1985). Measurements of conduit vessel blood flow during moderate exercise have demonstrated a two-phase blood flow response composed of a fast component attributed to mechanical factors, and a slower second phase driven by multiple agents produced or diminished during elevated aerobic metabolism (Shoemaker and Hughson, 1999; VanTeeffelen and Segal, 2006). While the full contribution of metabolic products to blood flow responses and their underlying mechanisms have not been fully elucidated, oxygen and carbon dioxide have long been understood to have independent vasoactive properties which act in a concentration dependent manner to increase blood supply during exercise. Indeed, there is evidence for multiple mechanisms governing vasoactive responses for both oxygen and carbon dioxide, though there is little data in the literature that describes the dynamics of these mechanisms or the overall time course of the resulting change in blood flow (reviewed in Jackson, 2016). To address this gap in our understanding, we quantified the microvascular blood flow responses to direct step changes in skeletal muscle PO_2_ and PCO_2_ in the absence of muscular contractions or other changes in aerobic metabolism. For the purposes of this discussion, we focus primarily on salient responses that are comparable with the decrease in skeletal muscle [O_2_] and increase in [CO_2_] seen at the onset of exercise and electrically stimulated contractions.

### Oxygen challenges

In this study we manipulated muscle oxygen concentration using a microfluidic gas exchange chamber that was directly interfaced with the EDL muscle *via* a gas permeable membrane. As expected, step changes in gas exchange chamber [O_2_] provoked rapid and profound alterations in capillary RBC SO_2_ that provides essential insight into the dynamics of our experimental method and important context for the resulting capillary blood flow responses (Figure 1). In each of the 4 oxygen challenges employed, significant changes in RBC SO_2_ were observed within 1–2 s of the step-change within the chamber with times to new steady state SO_2_ conditions ranging from 5—9 s. It is important to note that the dynamics of this imposed change in SO_2_ [*τ* = 1.4 s, for 7–2% (O_2_) challenge] is much faster than reported fast component decreases in microvascular (*τ* = 9.0 s) and interstitial (*τ* = 12.8 s) PO_2_ at the onset of electrically stimulated contractions using similar rat models (Behnke et al., 2001; Hirai et al., 2018). Step changes in chamber [O_2_] from 7–2% provoked a 52% peak increase in capillary RBC velocity, an 83% increase in RBC supply rate, and a 30% increase in capillary hematocrit over the course of the 2 min challenge, each of which are remarkably similar to previous observations of capillary hemodynamics during 1 Hz stimulated contractions in rat muscle (Kindig et al., 2002). The determined time transients of capillary RBC velocity (*τ* = 35.5 s), hematocrit (*τ* = 32.4 s), and supply rate (*τ* = 42.0 s) responses to 7–2% [O_2_] in our model were all of similar time scales and notably slower than responses during muscle contraction, particularly with respect to reported contraction induced supply rate response in animals (*τ* = 16 s) (Kindig et al., 2002; Poole et al., 2021) and early phase flow response in humans (*τ* < 7 s) (Shoemaker and Hughson, 1999). The discrepancy in dynamics with our model is almost certainly due to the absence of contraction that drives the early phase of exercise hyperemia via mechanical factors and rapid onset vasodilation (Tschakovsky et al., 1996; Laughlin et al., 1999; Mihok and Murrant, 2004; VanTeeffelen and Segal, 2006). Transient changes in capillary hematocrit in the present study suggest involvement of higher order arterioles that would be capable of affecting downstream hematocrit based on the Fåhræus-Lindqvist effect as also suggested by Kindig et al. (Fåhræus and Lindqvist, 1931; Barbee and Cokelet, 1971; Pries et al., 1986; Kindig et al., 2002). Further, in our model it is also likely that hematocrit and lineal density changes are driven, at least in part, by asymmetries in blood flow distribution between upstream arteriolar branches that supply the muscle volume influenced by our imposed gas perturbations, compared to other regions of the muscle which remain at basal conditions (Pries et al., 1989). Indeed, the hemodynamic changes observed during the 7–2% [O_2_] challenge likely integrate dilation of multiple levels of the arteriolar tree from terminal arterioles to higher order vessels *via* conducted signaling (reviewed in Bagher and Segal, 2011).

It is interesting to note that the dynamics of on and off transient responses to 12% and 2% oxygen challenges were not symmetrical, with faster capillary RBC velocity kinetics determined for 7–12% [O_2_] (*τ* = 7.0 s) and 2–7% [O_2_] (*τ* = 13.4 s) challenges (Table 2). However, the slower velocity dynamics based on the exponential fit to the 7–2% challenge does not describe the apparent multi-exponential nature of the response that includes a fast component evident over the first 20 s of the step change, and followed by a slower component that persists over the remainder of the 2 min challenge (Figure 2C). Similarly, this fast component is visible in the first 20 s of the velocity response to the 2–7% [O_2_] challenge (Figure 2D) and over the same time period in the 7–2% [O_2_] portion of the combined [O_2_] and [CO_2_] challenge (Figure 11B). The fast component of the velocity response to these challenges supports the role of rapid conducted signaling in oxygen mediated blood flow regulation, and provides further evidence of multiple mechanisms with distinct kinetics that overlap in time to produce the observed response. In contrast, the kinetics of hematocrit and lineal density responses were well represented by mono-exponential fits, and in the case of the 2–7% [O_2_] challenge showed profoundly slower dynamics (*τ* = 74.75 s) compared to the velocity response, with hematocrit changes likely driven by higher order arterioles as explained above.

### Carbon dioxide challenges

Step changes in the gas exchange chamber [CO_2_] provoked robust blood flow responses in each of the 4 challenge sequences studied. Increasing [CO_2_] from the putative resting concentration in the 5–10% challenge resulted in a 58% peak increase in capillary RBC velocity, a 16% increase in capillary hematocrit, and a 63% increase in capillary RBC supply rate which were similar in proportion to the increases seen over 2 min during the 7–2% [O_2_] challenges. The dynamics of the 5–10% [CO_2_] capillary hemodynamic responses were markedly slower than other CO_2_ challenges studied. Capillary RBC velocity (*τ* = 79.3 s), hematocrit (*τ* = 65.9), and supply rate (*τ* = 88.1 s) demonstrated distinctly slower kinetics that were two-fold longer than the oxygen mediated responses in the 7–2% [O_2_] challenge. Charter et al. reported similarly slow dynamics *in vivo* in second order cremaster arterioles when exposed to superfusate equilibrated with 5 and 10% CO_2_ (with measured PCO_2_ = 43 and 62 mmHg respectively) which showed vasodilation that progressively increased throughout the 2-min observation period at each concentration (Charter et al., 2018). Importantly, micropipette application of buffer equilibrated to the 5 and 10% [CO_2_] in the same study failed to elicit conducted vasodilation when applied to downstream capillaries or arterioles (Charter et al., 2018). The slow vascular response to high CO_2_ coupled with the progressive increase in venous PCO_2_ over the first 4 min of exercise (Casaburi et al., 1989) likely contributes to the slow phase of blood flow responses during this period.

Surprisingly, the strongest responses to step changes in [CO_2_] were observed in the 5–0% and 0–5% [CO_2_] challenges. The transition from 5–0% [CO_2_] provoked a 71% decrease in capillary RBC velocity over the 2 min challenge with a rapid time constant (*τ* = 18.9 s) both of which were highly symmetrical with the 0–5% [CO_2_] challenge (Table 4; Figures 7A,B). Interestingly, although the magnitude of capillary hematocrit responses were very similar (Table 4), the time constant for the 0–5% [CO_2_] challenge was more than two-fold longer (*τ* = 38.50 s) compared to the 5–0% [CO_2_] transition (*τ* = 15.3 s). It is unclear why there is such a discrepancy between the on and off transient dynamics associated with 0% [CO_2_], though it may be a product of the very low flow state during the first minute of the 0–5% [CO_2_] challenge. It could be argued that examination of responses to 0% CO_2_ are non-physiological; however, we expect that tissue PCO_2_ during these challenges to be non-zero due to the gradient between the gas exchange chamber and the muscle where mean PCO_2_ at a distance within the muscle volume is assumed to be ∼38 mmHg. Regardless, the profound decrease in blood flow observed under 0% CO_2_ indicates strong arteriolar reactivity to these conditions, though it is unclear whether or not the underlying mechanisms overlap with vasodilatory pathways at play when tissue [CO_2_] is increased beyond 5%.

Imposed changes in [CO_2_] caused a rapid shift in capillary RBC SO_2_ that serendipitously provides an indirect reference for the time course of tissue [CO_2_] changes (Figure 6), which followed a similar time scale to those observed when manipulating oxygen with the *τ* of SO_2_ changes ranging from 0.8 s in the 5–0% [CO_2_] challenge to 5.3 s in the transition from 10–5% (Table 4). We expect that this [CO_2_] dependent change in RBC SO_2_ reflects a shift in the oxy-hemoglobin dissociation curve caused by allosteric interactions *via* the Bohr effect that result in conformational changes in hemoglobin molecules associated with both [CO_2_] and the resulting change in pH. Based on the model by Dash and Bassingthwaighte, in the absence of pH changes we would expect RBC SO_2_ to only vary by 1% between [CO_2_] of 0–5%, while a change in [CO_2_] from 5–10% would be expected to cause a 3% shift in RBC SO_2_ (Dash and Bassingthwaighte, 2010). By comparison these findings suggest that the observed 5–6% shift in RBC SO_2_ during CO_2_ challenges is driven in part by a change in tissue pH. The apparent change in pH is notable as previous work has shown that the vasoactive effects of CO_2_ are through [CO_2_] in and of itself, and *via* the resulting change in [H^+^] (Charter et al., 2018). Unfortunately, in the current study we lacked the means to measure tissue pH within our muscle preparation, or at the interface with the exchange chamber. Additionally, SO_2_ measurements were somewhat confounded during 0% [CO_2_] conditions due to the extremely low RBC supply rates (Figures 10A,B), with many capillaries experiencing close to zero RBC supply. As we maintain the same plane of focus during data acquisition, and our method requires in focus capillaries with flowing RBCs to measure SO_2_, the low RBC supply rates resulted in higher variability due to the sparse number of SO_2_ measurements under this 0% [CO_2_] condition (Figures 6A,B).

### Combined O_2_ and CO_2_ challenges

In order to study the combined effects of oxygen and carbon dioxide mediated blood flow responses we examined a step change in gas exchange chamber [O_2_] from 7–2% for 1 minute, followed by an additional step change in chamber [CO_2_] from 5–10% (Figure 11). Over the minute following the step change in chamber [O_2_] from 7–2%, peak capillary RBC velocity increased by 25% with similar, though faster, dynamics (*τ* = 23.3 s) compared to the same step change in the non-combined 7–2% [O_2_] challenge described above (*τ* = 35.5 s). Similarly, capillary hematocrit increased by 15% over the 1-min period with somewhat faster dynamics (*τ* = 21.5 s) compared to the non-combined 7–2% [O_2_] challenge (*τ* = 32.42 s). Capillary RBC supply rate increased by 40% over the 7–2% [O_2_] portion of the combined challenge compared to the baseline period. The addition of a 5–10% [CO_2_] step change caused a peak increase in capillary RBC velocity by a further 78% above baseline over the 2-min combined challenge. Peak capillary hematocrit response showed a small 9% increase over baseline levels, while capillary RBC supply rate increased a further 76% over baseline by the end of the 5–10% [CO_2_] portion of the combined challenge. The time constant for the velocity response for the 5–10% [CO_2_] portion of the combined challenge (*τ* = 85.8 s) was consistent with the dynamics from the non-combined 5–10% [CO_2_] challenge (*τ* = 79.3 s). These findings clearly illustrate that the oxygen and carbon dioxide mediated blood flow responses to challenges that mimic conditions in exercise, are additive and operate with distinctly different dynamics.

Exponential fit for the capillary RBC SO_2_ changes over the course of the combined challenge following the 7–2% [O_2_] showed similar rapid dynamics (*τ* = 0.7 s) to the non-combined 7–2% [O_2_] challenge (*τ* = 1.4 s). The 7–2% [O_2_] step change abruptly reduced capillary RBC SO_2_ from 66% to a nadir of 41%, similar to that observed during the non-combined 7–2% [O_2_]. Similarly, as was observed in the non-combined 7–2% [O_2_] challenge there was an upwards drift in capillary RBC SO_2_ beginning ∼20 s after the step change that increased mean SO_2_ to 47%. We expect this partial restoration of capillary RBC SO_2_ is due to the flow response where increased oxygen supply *via* higher RBC supply rate partially restores tissue PO_2_ (Figures 1C, 11A). Furthermore, following the 5–10% [CO_2_] portion of the combined challenge, SO_2_ continued to rise to 56% as capillary RBC velocity and supply rate progressively increased over the remainder of the collection period. Again, we expect that this upwards drift in SO_2_ to reflect a partial restoration of tissue PO_2_ resulting from increased oxygen supply *via* the flow response. Interestingly, the rapid drop in SO_2_ that was noted in the non-combined 5–10% [CO_2_] challenge that reflects a shift in the oxy-hemoglobin dissociation curve as described above, was not observed during the combined response, perhaps due to the already elevated flow state.

This approach used to study the dynamics of O_2_ and CO_2_ as a mimetic for exercise has some limitations. [O_2_] and [CO_2_] challenges for each field of view were completed in a fixed sequence as described above in order to limit the apparent hysteresis that was noted during pilot studies that caused an upward drift in basal blood flow state following repeated exposure of the muscle to high [CO_2_]. While not ideal, this sequence of challenges was deliberately chosen as a means to mitigate the effect of this hysteresis on blood flow responses; future studies could randomize the order of perturbations and limit observations to a single series of challenges per animal. Furthermore, following analysis of our experimental data we noted that in the case of the 10–5% [CO_2_] challenge, there appears to not have been sufficient time to fully reach steady state during the baseline period at 10% [CO_2_] (see panel D in Figures 7, 10). Future studies should extend the pre-baseline period to 5 min at the desired gas concentrations to ensure a stable baseline is established. Lastly, these experiments were conducted under resting conditions in anesthetized animals where the muscle was not contracted but rather exposed to precisely controlled gas conditions mimicking the low [O_2_] and high [CO_2_] environment that would be expected during exercise. The conditions under which our data were collected, and the absence of muscular contraction should be considered when interpreting our findings in the context of exercise.

In summary, we have provided a thorough quantification of the magnitude and dynamics of oxygen and carbon dioxide mediated blood flow responses *in vivo* following abrupt step changes in tissue [O_2_] and [CO_2_], imposed via a microfluidic gas exchange chamber. Importantly, we demonstrate that in several cases the dynamics of hemodynamic responses in terms of capillary RBC velocity, lineal density, hematocrit, and supply rate that differ in time, show elements of concentration dependence, and in some instances are not symmetrical for on and off transient responses. Furthermore, we clearly show that combined [O_2_] and [CO_2_] perturbations elicit additive microvascular hemodynamic responses with each agent contributing different magnitudes and dynamics to the overall change in blood flow. For future studies our method presents an opportunity to study how adaptations in the context of exercise training, aging, and disease models may alter the dynamics of microvascular responses to skeletal muscle [O_2_] and [CO_2_]. Finally, our approach can be extended to examine how specific mechanisms contribute to the dynamics of oxygen and carbon dioxide mediated responses, which presents exciting additional avenues for future mechanistic studies.

## Data Availability

The data supporting the conclusions of this article will be made available by the authors, without undue reservation.
